# On‐Chip Metamaterial‐Enhanced Mid‐Infrared Photodetectors with Built‐In Encryption Features

**DOI:** 10.1002/advs.202415518

**Published:** 2025-01-10

**Authors:** Shicong Hou, Li Han, Shi Zhang, Libo Zhang, Kaixuan Zhang, Kening Xiao, Yao Yang, Yunduo Zhang, Yuanfeng Wen, Wenqi Mo, Yiran Tan, Yifan Yao, Jiale He, Weiwei Tang, Xuguang Guo, Yiming Zhu, Xiaoshuang Chen

**Affiliations:** ^1^ Shanghai Key Laboratory of Modern Optical Systems Terahertz Technology Innovation Research Institute and Engineering Research Center of Optical Instrument and System Ministry of Education University of Shanghai for Science and Technology 516 Jungong Road Shanghai 200093 China; ^2^ College of Physics and Optoelectronic Engineering Hangzhou Institute for Advanced Study University of Chinese Academy of Sciences No. 1, Sub‐Lane Xiangshan, Xihu District Hangzhou 310024 China; ^3^ College of Optical and Electronic Technology China Jiliang University Hangzhou 310018 China; ^4^ State Key Laboratory of Infrared Physics Shanghai Institute of Technical Physics Chinese Academy of Sciences 500 Yu‐Tian Road Shanghai 200083 China; ^5^ College of Physics and Optoelectronic Engineering Shenzhen University Shenzhen 518060 China

**Keywords:** infrared‐encrypted communication, mid‐infrared photodetector, optoelectronic logic computing, plasmonic microstructure, self‐powered photodetection

## Abstract

The integration of mid‐infrared (MIR) photodetectors with built‐in encryption capabilities holds immense promise for advancing secure communications in decentralized networks and compact sensing systems. However, achieving high sensitivity, self‐powered operation, and reliable performance at room temperature within a miniaturized form factor remains a formidable challenge, largely due to constraints in MIR light absorption and the intricacies of embedding encryption at the device level. Here, a novel on‐chip metamaterial‐enhanced, 2D tantalum nickel selenide (Ta₂NiSe₅)‐based photodetector, meticulously designed with a custom‐engineered plasmonic resonance microstructure to achieve self‐powered photodetection in the nanoampere range is unveiled. Gold cross‐shaped resonators are demonstrated that generate plasmon‐induced ultrahot electrons, significantly enhancing the absorption of MIR photons with energies far below the bandgap and boosting electron thermalization in Ta₂NiSe₅, yielding a 0.1 V bias responsivity of 47 mA/W—an order of magnitude higher than previously reported values. Furthermore, the implementation of six reconfigurable optoelectronic logic computing (“AND”, “OR”, “NAND”, “NOR”, “XOR”, and “XNOR”) are illustrated via tailored optical and electrical input‐output configurations, thereby establishing a platform for real‐time infrared‐encrypted communication. This work pioneers a new direction in secure MIR communications, advancing the development of high‐performance, encryption‐capable photonic systems.

## Graphical abstract

The Ta₂NiSe₅‐based plasmon‐enhanced self‐powered photodetector operates in the mid‐infrared (MIR) regime and incorporates a hybrid architecture comprising cross‐shaped plasmonic resonators integrated with a Ta₂NiSe₅ nanosheet. Through the precise design of plasmonic nanostructures combined with asymmetric microstructures, the system achieves a significantly enhanced MIR photoresponse while enabling real‐time optoelectronic logic computations. Additionally, it demonstrates seamless integration of computational functions and secure data transmission, exemplified by encrypted ASCII‐coded communication and encoded image transmission applications.

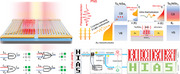

## 1 Introduction

The rapid proliferation of artificial intelligence (AI) has catalyzed a dramatic increase in optical sensory nodes, generating vast amounts of unstructured data across various wavelengths.^[^ ^1^, ^2^, ^3^ ^]^ While the visible spectrum dominates machine vision for intelligent systems, the invisible mid‐infrared (MIR) region, ranging from their ability to penetrate atmospheric obscurants to their sensitivity to thermal radiation, has emerged as crucial for advanced machine perception in applications such as thermal imaging, environmental sensing, medical diagnostics, and security monitoring.^[^ ^4^, ^5^, ^6^, ^7^ ^]^ MIR optical communication, in particular, offers substantial advantages over traditional microwave systems, including higher data transmission rates, greater immunity to interference, enhanced security, and broader spectral bandwidth.^[^ ^8^, ^9^, ^10^, ^11^, ^12^ ^]^ However, realizing the full potential of MIR communication and sensing systems necessitates overcoming significant technological challenges, as developing integrated, high‐performance systems for secure MIR communication demands innovations that transcend conventional photodetection approaches. At the heart of these innovations is the integration of reconfigurable optoelectronic logic computing (OELC) into photodetectors, which perform fundamental logical operations such as AND, OR, and NOT,^[^ ^13^, ^14^, ^15^, ^16^ ^]^ enabling on‐chip signal processing and encryption capabilities. In the MIR frequency range, OELC, despite their potential for ultrafast and parallel data processing in optical computing, face significant challenges that stem from both material limitations and device architecture constraints. One of the primary issues is that traditional semiconductors, such as silicon, exhibit poor absorption in the MIR range, limiting their effectiveness in MIR optoelectronic applications.^[^ ^17^ ^]^ Additionally, integrating MIR‐active materials, such as c(InSb) and mercury cadmium telluride (HgCdTe), with conventional electronic platforms is difficult due to lattice mismatches and thermal expansion coefficients,^[^ ^18^, ^19^ ^]^ leading to defects and degraded device performance. Furthermore, the efficient confinement and manipulation of MIR light within miniaturized devices is a significant challenge due to the longer wavelengths.

Addressing the inherent limitations of conventional MIR photodetectors based on 2D materials necessitates the development of a new class of devices that not only enhance MIR photon absorption but also integrate advanced functionalities, such as on‐chip encryption and optical logic operations, within the detection framework. The imperative lies in the development of a MIR photodetector exhibiting characteristics such as heightened responsivity, exceptional sensitivity, low noise levels, stability at room temperature, and compatibility with modern semiconductor platforms. In addition to the low energy of MIR photons, which often falls below the bandgap of 2D materials and leads to inefficient absorption and limited photoresponse, many single‐layer and few‐layer 2D materials, despite their promising electronic and optical properties, inherently exhibit low absorption rates due to their atomic‐scale thickness (2.3% for graphene, ≈2–5% for transition metal dichalcogenides^[^ ^20^, ^21^ ^]^). Overcoming this requires innovative material systems and device architectures that transcend traditional bandgap and absorption constraints. Recent advances in plasmon‐induced hot electron generation offer a promising pathway: By harnessing surface plasmon resonance (SPR),^[^ ^22^ ^]^ these devices effectively convert MIR photons into electrical signals, circumventing the conventional limitations imposed by semiconductor bandgaps.^[^ ^23^ ^]^ SPR, which occurs in nanostructured metallic materials, can amplify local electromagnetic fields by a factor of 10^3^ to 10⁵, dramatically increasing light absorption even for photons with energies far below the semiconductor bandgap. Recent studies have shown that gold nanostructures enhance light absorption in the MIR region by more than 90% through plasmonic coupling,^[^ ^24^ ^]^ enabling detection of a wide spectral band from 1.4 to 4.2 µm wavelength range.^[^ ^25^ ^]^ The rapid generation and transfer of hot electrons, occurring on a sub‐100 fs timescale,^[^ ^26^ ^]^ competes effectively with carrier recombination and energy relaxation processes, thereby minimizing losses and ensuring ultrafast response times.^[^ ^27^, ^28^ ^]^ This approach enables zero‐bias responsivities in the range of tens to hundreds of V/W,^[^ ^29^ ^]^ far surpassing the performance of traditional MIR photodetectors. Moreover, the ability to tailor the plasmonic response through nanostructure design provides an additional degree of freedom, allowing for the seamless integration of advanced functionalities, such as optical encryption and logic operations. This represents a paradigm shift in MIR photodetection, offering a pathway toward devices that combine high efficiency, speed, and security for next‐generation infrared sensing and communication technologies.

In this work, we propose a novel on‐chip metamaterial‐enhanced MIR photodetector with built‐in encryption capabilities, addressing critical limitations in current systems by combining high sensitivity, secure data handling, and logic functionalities at the device level. While Ta_2_NiSe_5_ has shown promising photodetection capabilities across the visible to MIR spectrum,^[^ ^30^, ^31^, ^32^, ^33^ ^]^ its performance in the MIR band remains suboptimal due to limited absorption and slow carrier dynamics. Our innovative approach incorporates a plasmonic metastructure, precisely engineered in an asymmetric configuration with an interdigital design, to dramatically enhance MIR absorption and responsivity. This hybrid configuration facilitates self‐powered operation by harnessing plasmonic hot electron generation, achieving a responsivity that surpasses conventional detectors by over an order of magnitude. Furthermore, this system enables the execution of logical operations—such as AND, OR, and XOR operations—through the strategic interplay of optical and electrical inputs, offering a new platform for secure, real‐time data encryption within MIR communication channels. By integrating this photodetector into an optical communication network, we demonstrate the successful transmission of encrypted “HIAS” information, underscoring the device's potential for next‐generation secure, high‐speed MIR communication systems. Our study not only offers a robust solution for the advancement of high‐performance self‐powered infrared photodetectors but also paves the way for the realization of efficient information transmission within infrared optical communication systems.

## 2 Results and Discussion

**Figure 1:**
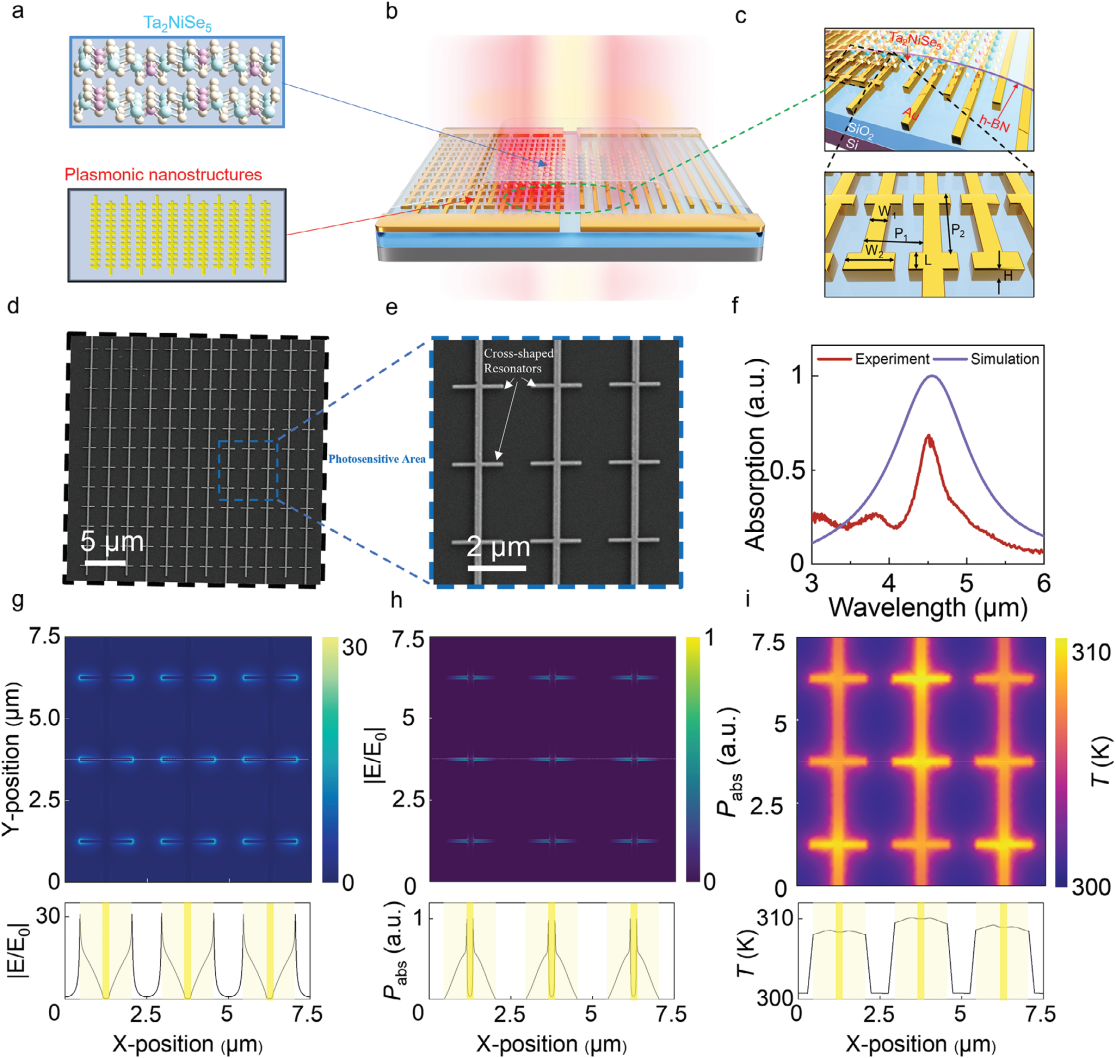
Design of plasmon‐excited MIR detectors. a) Diagram of Ta_2_NiSe_5_ atomic structure and plasmonic nanostructures. b) The overall structure of the metamaterial device exhibits different light absorption under MIR radiation. c) Visualization of the device architecture and dimension parameters. The photodetector consists of a Si/SiO_2_ substrate, an asymmetric Au nanostructure, Ta_2_NiSe_5_ photoresponsive material, and a h‐BN protective layer. d,e) The scanning electron micrographs of the proposed plasmonic nanostructures at different magnifications. Figure e shows 3 × 3 resonant units. Scale bars are 5 µm d), 2 µm e). f) Simulated and measured spectral absorption (blue and red line), The laser power is set to be 200 µW. g–i) The simulated electrical, optical, and thermal behavior for the same 3 × 3 unit cells. The electromagnetic field distribution is illustrated in g), the spatial absorption distribution is illustrated in h) and the thermal distribution is shown in i). The cross sections along the center line (*y* = 3.75 µm) for each simulation type (g–i) are presented at the bottom of each panel.

The metamaterial‐enhanced mid‐infrared (MIR) photodetector in **Figure** **1**a–c, features a hybrid architecture composed of cross‐shaped plasmonic resonators integrated with a Ta₂NiSe₅ nanosheet, layered on a SiO_2_/ p^+^‐Si substrate, in which a hexagonal boron nitride (h‐BN) passivation layer provides enhanced device stability. This architecture leverages plasmonic field confinement, with the cross‐shaped resonators engineered to amplify the interaction between incident MIR light and the Ta₂NiSe₅ active layer. The grating structure, optimized for a width of *W*_1_ = 0.2 µm, thickness *H* = 60 nm, and periodicity *P*_1_ = 2.5 µm, enables resonant coupling, effectively boosting the absorption of sub‐bandgap photons. The optical micrographs and scanning electron microscopy (SEM) images in Figure S3a,b (Supporting Information) and Figure 1d,e reveal the uniformity and precision of the nanostructure fabrication. The total active area of the device, measuring 30 µm × 30 µm, consists of a 12 × 12 array of resonant units. Each resonant unit, as highlighted in Figure 1d, measures 2.5 µm × 2.5 µm, designed to optimize electromagnetic field localization. The intricate design of these plasmonic resonators enables significant enhancement in MIR absorption by concentrating the electric field near the Ta₂NiSe₅ layer, facilitating efficient hot‐electron generation.

To investigate the optical absorption characteristics of the designed plasmonic structure, finite difference time domain (FDTD) simulations are performed, optimizing parameters such as wavelength, structure size, and polarization angle (see Figure S1, Supporting Information). The plasmonic nanostructure can reach the optical absorption peak at the designed 4600 nm mid‐infrared wavelength by simulation, as shown in the blue curve in Figure 1f. It shows good consistency with the absorption spectrum (Figure 1f, red curve) measured by a Fourier transform infrared (FTIR) spectrometer coupled with a microscope. The explanation of The simulated absorption range is broader than that observed in experiments can be summarized as follows. First, the simulation model only accounts for radiation loss, whereas the actual material experiences a combination of radiation and non‐radiation loss, including ohmic loss, defect scattering, and other non‐radiation loss mechanisms. Second, the simulation employs a periodic boundary condition, which results in sustained oscillation and radiation of energy within the structure, leading to augmented radiation losses and an expansion of the resonance peaks. The discrepancy in absorption intensity could potentially be attributed to the precision of the equipment used in the fabrication process. The simulated electric field distribution under 4600 nm plane wave irradiation, as shown in Figure 1g–i, demonstrates clear plasmonic behavior characterized by dipole antenna‐like electric field enhancement. The spatial distribution of absorption is derived from the simulated electric field and is calculated theoretically using Poynting's method. The bottom panel of Figure 1g illustrates a cross‐sectional view along the central line of the marker (indicated by a white dotted line), further highlighting the concentrated electric field near the resonant unit. This is attributed to the localized surface plasmon resonance (LSPR) within the cross‐shaped resonators, which are longitudinally interconnected via an interdigital metal structure (Figure 1c). The design ensures minimal disruption to the overall field distribution while maintaining strong coupling between adjacent resonant units. The cross‐sectional view in Figure 1g–i further highlights the electric field concentration near the resonant unit, confirming the effectiveness of the structure in confining the electromagnetic energy. As shown in Figure 1g, absorption is tightly confined to the vicinity of the resonant unit, with the cross‐sectional diagram at the lower part of Figure 1g offering a clearer visualization of this localized effect. To efficiently extract the locally photoexcited hot carriers, Figure 1i shows the simulated temperature distribution across the 3 × 3 array of plasmonic resonators, revealing a significant temperature gradient resulting from localized absorption of MIR radiation. This temperature gradient, driven by hot carrier generation, plays a critical role in enhancing the overall photoresponse. The simulations, conducted at a room temperature of 300 K, account for radiation, convection, and heat conduction, offering a detailed understanding of the plasmon‐induced carrier dynamics under MIR illumination. Overall, the simulations indicate a strong overlap between regions of maximum electric field enhancement (Figure 1g) and areas of peak hot carrier generation (Figure 1i), suggesting that the plasmonic structure provides both efficient carrier excitation and short extraction pathways, promising fast and highly effective carrier collection.

**Figure 2:**
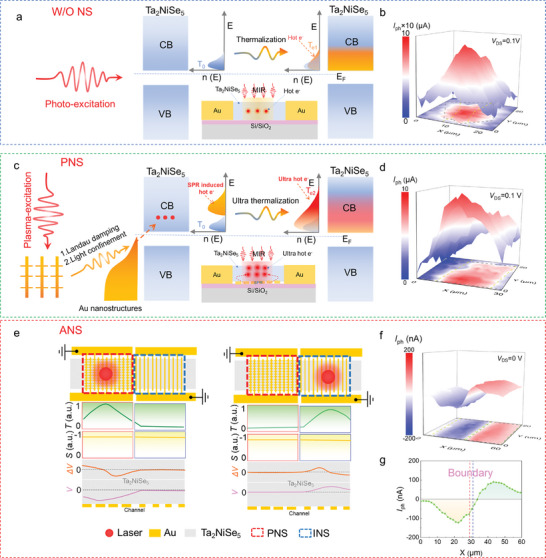
Plasmon‐enhanced Ta_2_NiSe_5_ MIR photoresponse mechanism investigation. a,c) The energy band and mechanism diagrams of the generation process of traditional hot electrons and plasmonic ultra hot carriers under infrared laser excitation are respectively shown in a,c). Here, *T*_1_, *T*_e1_, and *T*_e2_ represent the temperature of the phonon system, hot electrons, and ultrahot electrons, respectively. b,d) The scanning photocurrent mappings of W/O NS and PNS under 4600 nm laser at 0.1 V bias were measured in b) and d). e) Laser excitation‐induced temperature distribution *T*(x), Seebeck coefficient *S*(x), potential gradient Δ*V*, and the potential distribution *V* along the channel when the laser spot is located at the middle of PNS (left) and GNS (right). f) The scanning photocurrent mappings of ANS under 4600 nm laser at 0 V bias were measured. g) Line profile of the *I*_ph_ distribution cut along the center of the channel labeled by a green dashed line in f), showing an asymmetric distribution of photocurrent.

To elucidate the operational principle of plasmon‐enhanced Ta_2_NiSe_5_ MIR detectors, we compared devices with plasmonic nanostructures (PNS) or without any nanostructure (W/O NS) by analyzing their photoresponse. The morphology of both device types was confirmed via scanning electron microscopy (SEM) and energy‐dispersive spectroscopy (EDS), which confirmed the material composition as Ta, Ni, and Se (see Figure S7, Supporting Information). To assess the mid‐infrared (MIR) response distribution of the devices, scanning photocurrent mapping (SPCM) analysis using a 4600 nm laser was conducted on devices both with PNS and W/O NS, where the scanning areas correspond to the dotted box markings in Figure S6 (Supporting Information). The SPCM results, depicted in **Figure** **2**b,d, indicate that the peak MIR photoresponse detected in the PNS is ≈10 µA, which is an order of magnitude higher than that in the W/O NS device, attributed to improved light absorption facilitated by plasmonic effects. For the W/O NS device, the photon energy of 0.27 eV (corresponding to 4600 nm) is insufficient to overcome the intrinsic bandgap of Ta₂NiSe₅ (0.37 eV).^[^ ^34^ ^]^ Consequently, direct photogeneration of electron‐hole pairs is not possible. Instead, the absorbed photons undergo non‐radiative processes, transferring their energy via electron‐electron and electron‐phonon scattering to generate a non‐equilibrium distribution of hot electrons through a thermalization process.^[^ ^35^ ^]^ The hot carriers, described by a Fermi‐Dirac distribution,^[^ ^36^ ^]^ follow the expression:

(1) $$f\left(E\right)=\frac{1}{exp\left(-\frac{E-E_{F}}{kT_{e}}\right)+1}$$

where *k* is the Boltzmann constant, and *T*_e_ is the hot electron temperature (*T*_e_ > lattice temperature *T*_lattice_≈ environment temperature *T*_0_), it can be seen that the energy distribution is only determined by *T*_e_. The mechanisms behind the photoresponse of the traditional hot electron generation (W/O NS) and the plasmonic hot electron generation (PNS device) are presented in Figure 2a and c, respectively. In the case of W/O NS, hot electrons are generated by thermalization, resulting in an extended high‐energy tail in the electron distribution, contributing to the observed photocurrent. However, the limited absorption efficiency of 2D Ta₂NiSe₅ in the MIR range restricts the generation and distribution of these hot carriers. To overcome these limitations, we employ LSPR to surpass the intrinsic absorption and band gap constraints of Ta_2_NiSe_5_, thereby exciting ultra‐hot electrons, as illustrated in Figure S8 (Supporting Information).

As depicted in Figure 2c, the PNS induces resonant coupling with the incident MIR radiation, which leads to intense near‐field enhancement in the proximity of the Au nanostructures. This field localization is governed by the excitation of surface plasmons at the metal‐semiconductor interface, amplifying the electric field and boosting optical absorption far beyond the intrinsic capabilities of Ta₂NiSe₅. In addition to electromagnetic field enhancement, the non‐radiative decay of plasmons via Landau damping results in the generation of hot electrons that exhibit an extended high‐energy tail, significantly broader than the thermalized carriers in the W/O NS device. These ultra‐hot electrons are characterized by an electron temperature (*T*_e2_) much higher than the lattice temperature (*T*₀), as indicated in Figure 2c. Unlike traditional photoexcitation, these hot carriers are injected across the barrier at the Au/Ta₂NiSe₅ interface, which is much smaller than the semiconductor bandgap, facilitating efficient electron injection into the conduction band. Moreover, the PNS not only enhances absorption but also provides non‐radiative relaxation pathways that directly excite hot carriers, leading to a longer thermal tail and improved energy distribution.^[^ ^37^ ^]^ The enhanced photoelectric conversion efficiency of the PNS device arises from this dual effect of localized field enhancement and plasmon‐induced hot electron generation. The mechanism bypasses the limitations imposed by the bandgap, utilizing sub‐bandgap photon energies to excite and inject carriers efficiently. This is further illustrated in Figure 2c, where the broader high‐energy distribution of ultra‐hot electrons leads to a much higher photocurrent, as compared to the W/O NS device.

To further elucidate the enhancement effect of PNS and the role of the asymmetric nanostructure (ANS) in the photo thermoelectric (PTE) response, we measured the laser‐induced thermoelectric voltage distribution across the device. When the 4600 nm mid‐infrared laser irradiates the PNS, the localized plasmonic resonance significantly enhances the absorption of photons, leading to intense localized heating and a substantial temperature gradient within the Ta₂NiSe₅ layer. The localized heating drives the diffusion of non‐equilibrium hot carriers, which follow the thermal gradient, from the PNS region toward the grating structure (GNS). This diffusion generates a thermoelectric voltage (Δ*V*) due to the Seebeck effect, creating a potential difference between the two regions. Conversely, when the laser irradiates the right GNS, the hot carriers diffuse in the opposite direction—from the GNS to the PNS—reversing the sign of the thermoelectric signal. As depicted in Figure 2f, the zero‐bias photocurrent mapping (*I*_ph_, calculated as *I*_ph_ = *I*_illumination_−*I*_dark_) for the PNS device under 200 µW laser excitation reveals a significant asymmetry in the photocurrent distribution across the device. The peak *I*_ph_ values on the PNS and GNS sides can reach −120 nA and 90 nA, respectively, indicating a strong PTE response induced by the plasmonic‐enhanced thermal gradient. The line profile of the photocurrent, shown in Figure 2g, further illustrates the opposite signs of *I*_ph_ between the PNS (negative) and GNS (positive) regions, consistent with the directional diffusion of hot carriers. The shift of the photocurrent zero‐crossing toward the GNS side reflects the superior light absorption and heat generation efficiency of the PNS, which results in a more significant thermoelectric effect compared to the GNS. As observed in Figure 2f, the photocurrent is distributed across the entire device channel in correspondence with the temperature gradient, aligning with the characteristics of the PTE effect. This observation suggests that the PTE effect, driven by the laser‐induced temperature gradient, dominates the photocurrent response, with the PV contribution being minimal.

In this device configuration, the Seebeck coefficient (*S*)^[^ ^38^ ^]^ of Ta₂NiSe₅ is assumed to remain uniform across the device channel, implying that variations in the thermoelectric voltage primarily depend on the spatial distribution and magnitude of the temperature gradient induced by laser irradiation. According to thermodynamic principles, the photothermoelectric response can be understood as a manifestation of the non‐equilibrium thermodynamic state induced by continuous photoexcitation. Specifically, the incident mid‐infrared radiation creates localized heating, which in turn generates a non‐uniform temperature profile that disrupts thermal equilibrium within the material, leading to a differential thermopower potential across the channel. The Peltier effect,^[^ ^39^ ^]^ which can manifest as localized heating or cooling at the metal‐semiconductor interface due to non‐uniform current density, also plays a crucial role in defining the temperature profile in such plasmon‐enhanced configurations, further contributing to the spatial asymmetry observed in the photocurrent distribution. The spatial temperature distribution, as illustrated in Figure 2e, indicates that when the laser is centered on the PNS, the temperature gradient reaches its peak due to the localized field enhancement arising from LSPR, which facilitates energy dissipation from the excited plasmonic states into the electronic system via non‐radiative decay mechanisms such as Landau damping. This elevated electron temperature (*T*_e_), which significantly exceeds the ambient lattice temperature, extends the hot electron distribution and results in a greater number of carriers occupying high‐energy states, thus amplifying the thermoelectric effect. However, as the laser is shifted toward the GNS side, the efficiency of light absorption diminishes due to the lack of plasmonic field enhancement, consequently reducing the localized temperature at the laser spot center. This decrease in thermal energy dissipation leads to a less pronounced temperature differential (Δ*T*) along the channel, which is reflected in a reduced thermoelectric potential and lower resultant photocurrent. The localized increase in entropy, as dictated by the second law of thermodynamics, effectively drives the hot carriers from regions of higher temperature to regions of lower temperature, resulting in a net diffusion current that establishes the measurable thermoelectric voltage *V* = *∫*−*S*Δ*T*. Consequently, the enhancement of light absorption through plasmonic resonance and the conversion of absorbed photon energy into thermal energy are key mechanisms that contribute to a higher photothermoelectric conversion efficiency.

**Figure 3:**
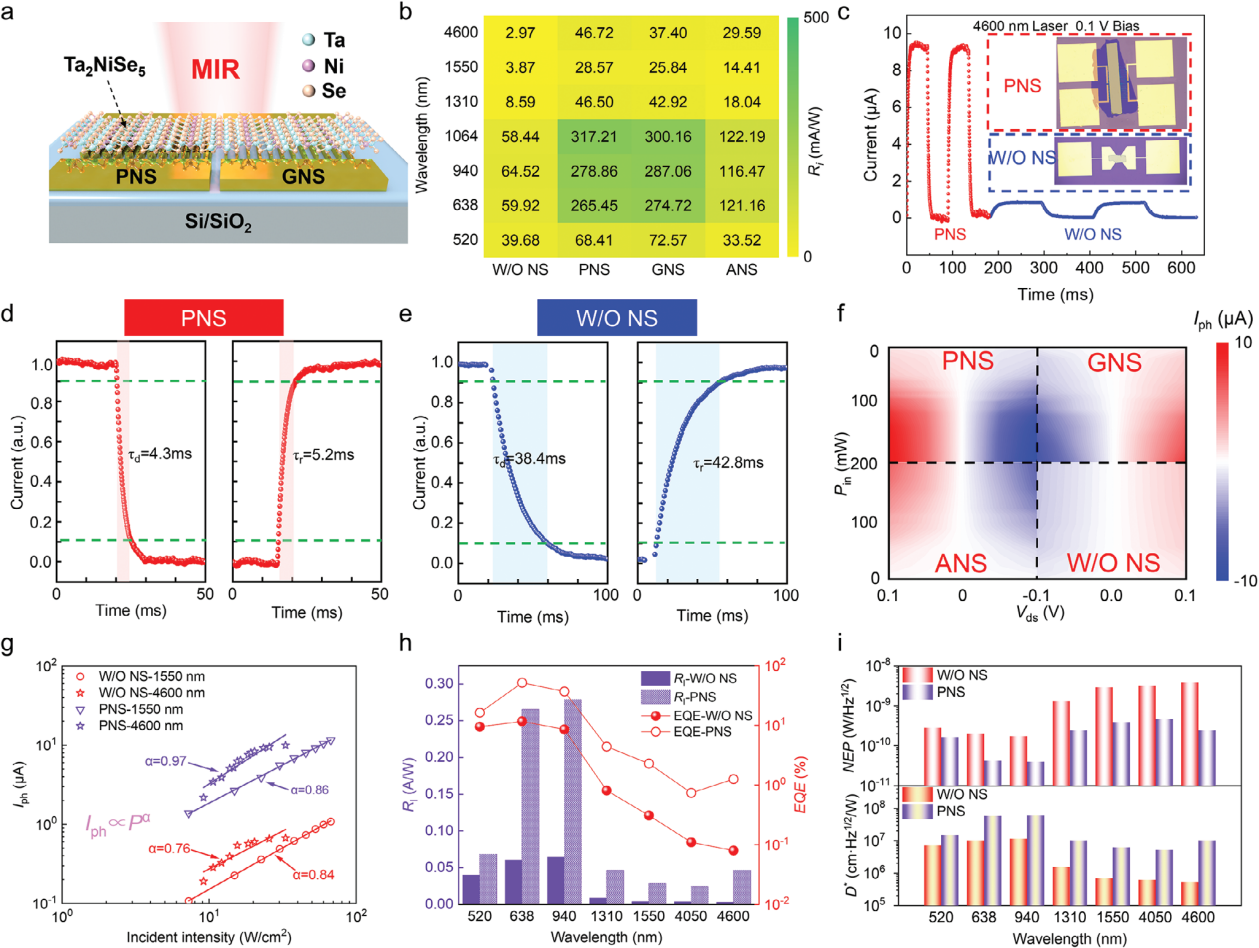
Photoelectric performances of Ta_2_NiSe_5_ based on different structures. a) Schematic of Ta_2_NiSe_5_ device based on plasmon‐enhanced asymmetric structure under MIR excitation. b) The photocurrent of different structures (W/O NS, PNS, GNS, and ANS) under a 0.1 V bias and with 100 µW incident power, spanning from visible to MIR band. c) Time‐resolved photocurrents of PNS and W/O NS at 4600 nm under 0.1v bias, illustrations are optical micrographs of PNS and W/O NS. d,e) The response speed of the PNS and W/O NS at 4600 nm under 0.1v bias. f) Source‐drain voltage (*V*_ds_) and incident power (*P*_in_) dependent photocurrent of four structures under 4600 nm laser extracted from the *I–t* curves. g) Measured photocurrent of the W/O NS and PNS under 1550 and 4600 nm laser as a function of incident intensity. h) Wavelength‐dependent responsivity and external quantum efficiency of the W/O NS and PNS for *V*_ds_ = 0 .1V. i) Noise equivalent power and specific detectivity as a function of different wavelengths at 0.1 V bias.

To fully demonstrate the advantages of Ta_2_NiSe_5_ photodetectors that utilize PNS in the MIR region, we conducted a comprehensive evaluation of various optoelectronic performance metrics across different device architectures and wavelength bands. **Figure** **3**a schematically illustrates the PNS and GNS‐based asymmetric nanostructure (ANS) configurations, where Ta₂NiSe₅ is deposited over gold nanostructures to enhance light‐matter interactions. The PNS is characterized by the presence of plasmonic dipoles enabling efficient MIR plasmon resonance, whereas the GNS exhibits weaker coupling effects. To quantitatively compare the photoresponse of different structures, as shown in Figure S9a–c (Supporting Information) (corresponding to PNS, GNS, and ANS, respectively), MIR scanning photocurrent mapping (SPCM) measurements were performed under a 0.1 V bias. As shown in Figure 3b, the responsivity (*Ri*) trends across wavelengths from 520 to 4600 nm reveal that PNS structures exhibit significantly enhanced photoresponse generation,^[^ ^40^ ^]^ particularly in the MIR range, where plasmonic effects dominate. Notably, the PNS maintains a higher photocurrent at 4600 nm compared to GNS and other configurations in Figure 3b. This enhancement is attributed to the stronger plasmonic field confinement and the efficient generation of hot carriers within the PNS structure, which sustains carrier excitation even at longer wavelengths. We further investioperationd the influence of external bias voltage and incident light power on the photoresponse stability (Figure 3f). The extracted photocurrent values under varying conditions indicate consistent and robust device performance, with PNS exhibiting a higher responsivity across all tested conditions. Figure 3g explores the power‐dependent photoresponse of the photodetectors under 0.1 V bias, where the relationship between the photocurrent (*I*_ph_) and the incident power (*P*) follows a power‐law dependence, *I*_ph_∝*P* ^α^, with the exponent α providing insight into the efficiency of photon utilization.^[^ ^41^ ^]^ Under 4600 nm illumination, the PNS device exhibits an α value of 0.97, which is remarkably close to the ideal value of 1. The linearity of the photoresponse is attributed to the plasmonic effect, which enhances the interaction between light and the material, alleviates nonlinear saturation effects, and optimizes both light absorption and charge carrier generation. The behavior is expected in plasmonic nanostructures, where LSPR leads to intense localized electromagnetic fields, enhancing the absorption cross‐section and facilitating efficient hot electron generation, even for lower‐energy photons in the MIR regime. In contrast, the W/O NS device, with α values of 0.76 and 0.84 under 4600 and 1550 nm illumination, respectively, shows a sublinear response, indicating a less efficient process for converting photons into usable charge carriers at longer wavelengths. The sublinear behavior is attributed to the absence of plasmonic enhancement and the limited generation of hot carriers in the W/O NS configuration, highlighting the critical role of plasmonic effects in optimizing MIR photoresponse.

The response time measurements of the PNS and W/O NS devices, captured using an additional lock‐in amplifier and oscilloscope in Figure 3c, further emphasize the impact of plasmonic enhancements on carrier dynamics. The PNS device exhibits rise and fall times of 4.3 and 5.2 ms, respectively (Figure 3d), which are an order of magnitude faster than the 38.4 and 42.4 ms recorded for the W/O NS device (Figure 3e). The dramatic improvement in response speed is indicative of the efficient generation and injection of hot electrons facilitated by the PNS structure. The sub‐picosecond hot electron generation process, driven by non‐radiative decay of plasmons via Landau damping,^[^ ^42^ ^]^ enables rapid carrier excitation and transport across the metal‐semiconductor interface, significantly reducing the temporal delay associated with traditional photoresponse mechanisms. However, the observed discrepancy between the response speed and the theoretical hot electron transfer times suggests that additional factors, such as interface defects, charge trapping, and carrier recombination, may still influence the overall response dynamics. Additionally, Figure 3h provides a detailed analysis of the responsivity (*R*_I_) and external quantum efficiency (*EQE*) of the PNS and W/O NS devices across the visible to mid‐infrared spectrum. At 4600 nm, the PNS device exhibits a responsivity of 47 mA W^−1^, approximately 16 times higher than the W/O NS device, which only achieves 3 mA W^−1^. This enhancement can be directly linked to the plasmon‐induced extension of the absorption bandwidth and the efficient generation of hot carriers. The corresponding *EQE* values show a similar trend, further corroborating the superior photon‐to‐electron conversion efficiency enabled by plasmonic enhancements, particularly in the sub‐bandgap MIR region, where conventional photodetection mechanisms are less effective. Finally, the noise equivalent power (*NEP*) and normalized detectivity (D*), key parameters for evaluating the sensitivity and noise characteristics of photodetectors. As shown in Figure 3i, the PNS device exhibits an *NEP* of 2.39 × 10⁻¹⁰ W Hz^−^¹^/^^2^ and a detective of 1.02 × 10⁷ cm·Hz^−^¹^/^^2^/W at 4600 nm, compared to the W/O NS device, which demonstrates significantly poorer performance with an NEP of 3.77 × 10⁻⁹ W Hz^−^¹^/^^2^ and a detectivity of 5.31 × 10⁵ cm·Hz¹^/^^2^ W^−1^. The reduction in *NEP* and corresponding increase in D* for the PNS device clearly indicate the advantages of plasmonic nanostructures in reducing noise and improving sensitivity, particularly in the MIR regime. These results underscore the ability of PNS to enhance weak light detection, driven by the efficient hot electron generation and collection enabled by the plasmonic effects. In addition, we believe that combining the PNS with a gold film to form a resonant cavity can further enhance the optoelectronic performance of the detector,^[^ ^43^ ^]^ which will be an important direction for our future work.

**Figure 4:**
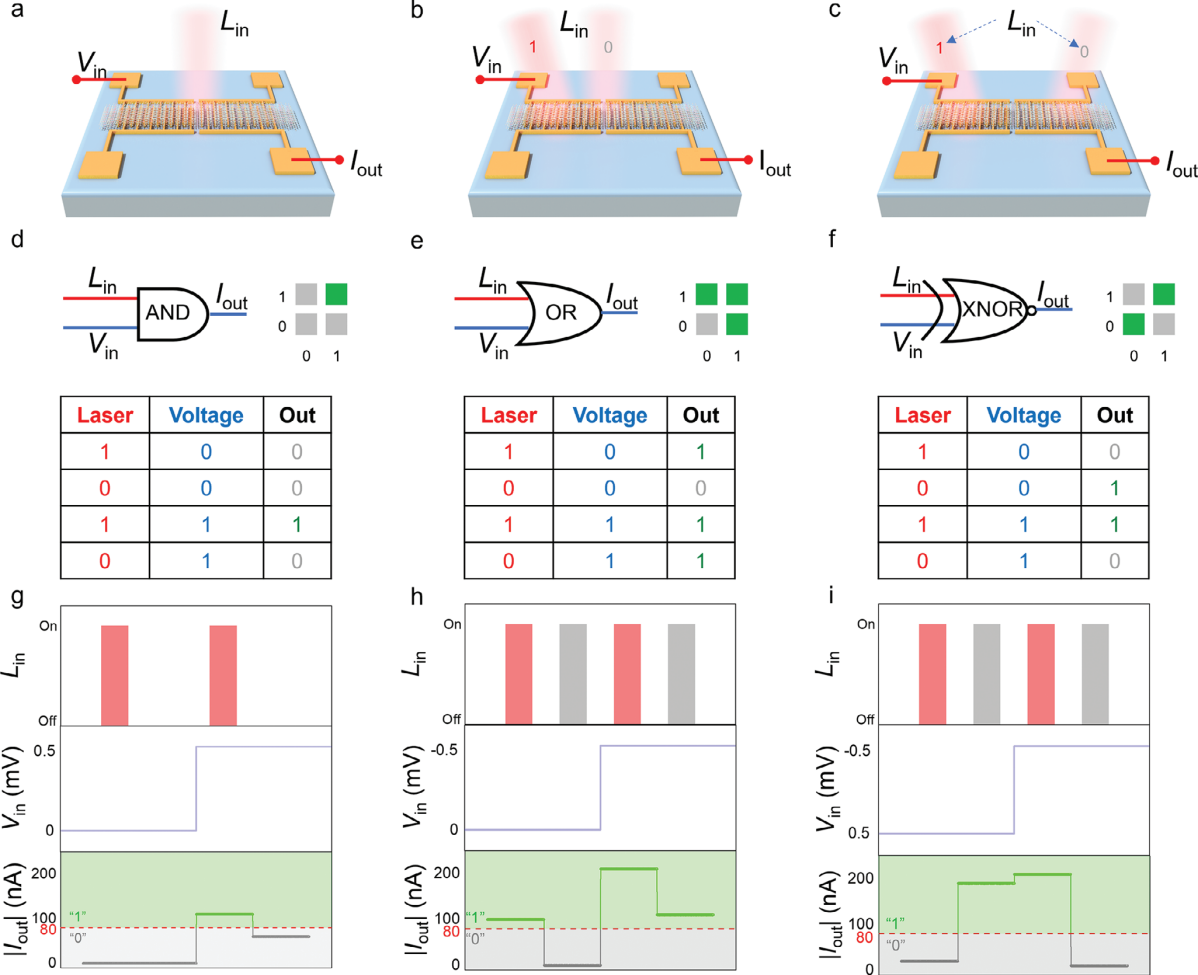
Characterization of MIR OELC based on PSPD. a–c) Schematics of the logic AND, OR, and XNOR operation system with mixed optical and electrical inputs. d–f) Symbolic schematics and truth tables of the logic AND, OR, and XNOR operation. g–i) The absolute value of the output current measured under the input sequence corresponding to the logic AND, OR, and XNOR operation.

Leveraging the plasmon‐enhanced self‐powered photodetector (PSPD), we propose a novel reconfigurable OELC system that is capable of performing six fundamental Boolean logic operations—“AND”, “OR”, “XNOR”, “XOR”, “NOR” and “NAND”—within a single, compact device framework, as depicted in **Figures** **4** and S14 (Supporting Information). This innovative system capitalizes on the synergistic modulation of both optical and electrical inputs to dynamically reconfigure the logic functions, thereby enabling real‐time operational flexibility. By exploiting the plasmonic enhancement of localized electromagnetic fields, which amplifies the light‐matter interaction and increases the generation of hot carriers, we demonstrate a logic architecture that can adapt its output response based on the spatiotemporal configuration of the incident laser and the applied bias voltage. This operational adaptability is fundamental to reconfigurable computing, where the processing unit can dynamically switch between different logic operations based on the incoming signals and the desired operation, thus allowing complex computation to be achieved with fewer physical resources. Figure S13a (Supporting Information) shows the output characteristics under dark and 4600 nm light, and after local amplification, four current states at zero bias are discussed in Figure S13b (Supporting Information): (1) When no photon is triggered, the current shows a relatively low level (yellow line). (2) When the laser irradiation causes the photon to be triggered at the center of the PNS or GNS, the current shows a high level (red line or blue line) due to the action of the photogenerated current. (3) When the laser is irradiated in the middle of PNS and GNS, a small number of directional carriers will be generated due to the asymmetry of the structure, resulting in a low current level (green line). Therefore, various OELCs can be realized in a single device by programming detection units with different light responses.

In Figure 4a, we illustrate the implementation of the AND logic operation, where the laser irradiation and the bias voltage serve as input1 (IN1) and input2 (IN2), respectively, while the resulting photocurrent functions as the output signal (OUT). The dynamic reconfigurability of the logic operation stems from the precise control of the optical operation (laser) and the electric operation (bias voltage), allowing for the modulation of the device response to different combinations of inputs. The operational truth table in Figure 4d presents the input and output states: the red and blue lines denote the optical and electrical signal inputs, while the green and gray squares indicate the “1” and “0” states of the output signal. Additionally, the logic truth table for the AND operation is depicted therein. The output current for the four input states of the logic AND operation is measured experimentally, as depicted in Figure 4g. The “1” and “0” states of IN1 correspond to the activation and deactivation of the MIR laser irradiated at the center of the ANS, respectively. The “1” and “0” states of IN2 correspond to biases of 0.5 mV and 0 mV, respectively. The absolute value of the current is elected as the output signal (OUT), with high (>80 nA) and low (<80 nA) absolute output currents representing OUT‐1 and OUT‐0, respectively. When the input signals are combined in a specific sequence IN‐00, IN‐01, IN‐10, and IN‐11as per the design, the device yields an output that adheres to AND logic operation. Furthermore, the inherent programmability of the device is demonstrated by the reconfiguration of the same hardware to realize an OR logic operation in Figure 4b,e. In this configuration, the IN1 values (“1” or “0”) are assigned to the laser irradiation at either the center of the PNS or ANS, while the IN2 values represent bias voltages of −0.5 and 0 mV, respectively. The output current is high (>80 nA) for all input combinations except IN00, where a low current is observed due to the minimal light absorption and reduced plasmonic enhancement in the ANS region. This switching behavior effectively constructs the OR operation, where the output is high if either of the inputs is high, demonstrating the capability to seamlessly switch between logic operations based on input conditions without requiring any physical alteration of the device structure. The adaptability of the system is further evidenced by its ability to implement more complex Boolean operations, such as the exclusive XOR operation, which is a nonlinear logic function that plays a fundamental role in computational circuits and encryption algorithms due to its capacity to differentiate between identical and non‐identical inputs.^[^ ^44^ ^]^ As shown in Figure 4c–f, the XOR operation is realized by utilizing the laser position over either the PNS or INS as IN1, while the bias configuration serves as IN2. IN1, with values of “1” and “0”, indicates the central position of laser irradiation over the PNS or GNS, respectively. Meanwhile “1” and “0” f or IN2 represent biases of −0.5 and 0.5 mV, respectively. Upon sending the sequence signals IN00, IN01, IN10, and IN11, the logic XNOR operation is implemented, as shown in Figure 4i.

As an extension of this reconfigurable system, we successfully implemented additional logic gates, including NOR, XNOR, and NAND, as depicted in Figure S14 (Supporting Information). These operations are critical in building complex logical expressions and combinational circuits, forming the basis of arithmetic operations and decision‐making processes in digital systems. The ability to implement all these fundamental logic operations within a single device highlights the potential of this system for use in adaptive logic networks, where devices must be capable of performing a wide range of computational tasks with minimal reconfiguration time. The plasmon‐enhanced light absorption and the optoelectronic tunability of this system provide a platform for future developments in intelligent sensing and secure communication, where real‐time reconfigurable logic is crucial for dynamic data processing and encryption.

**Figure 5:**
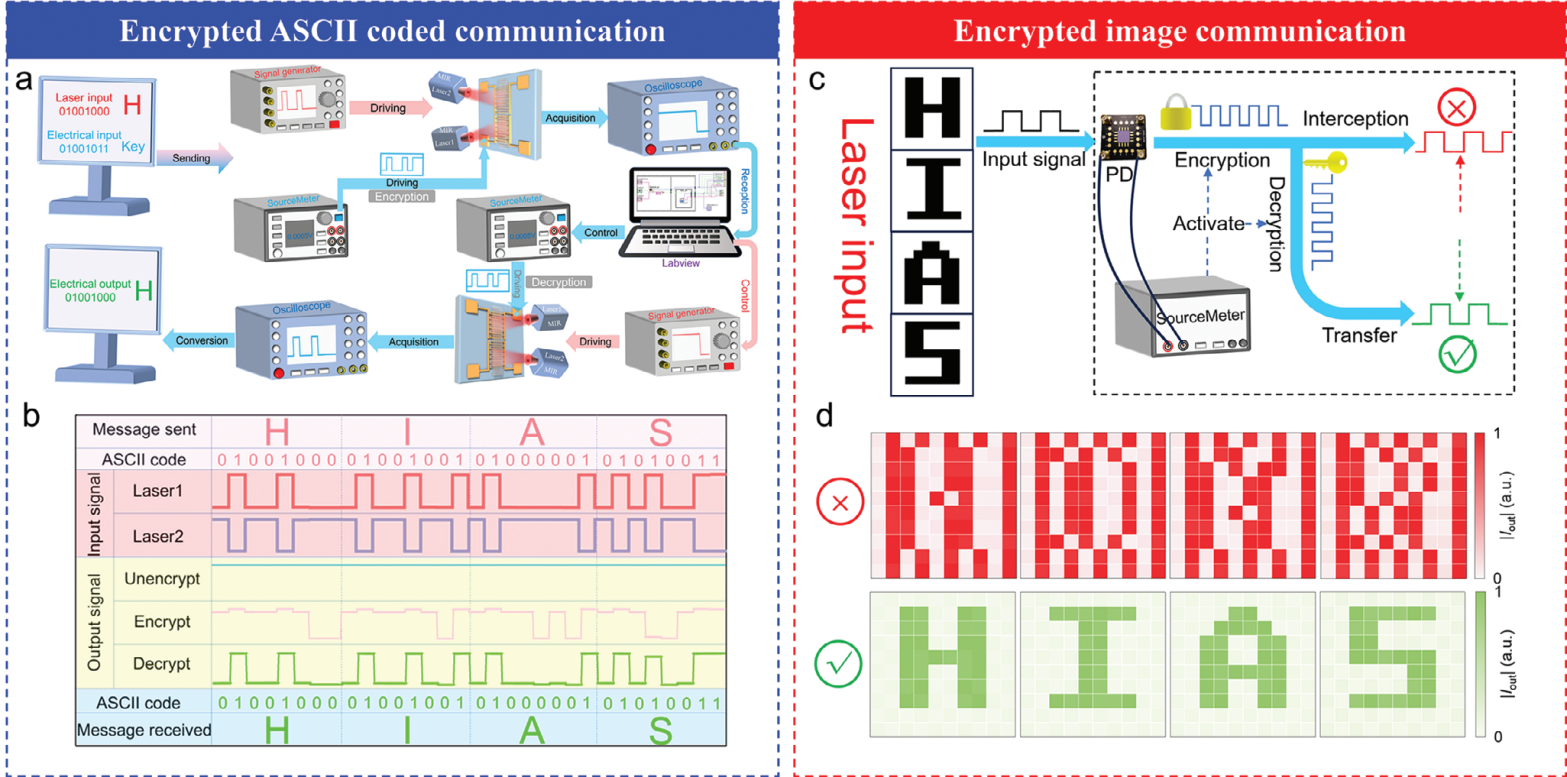
Encrypted ASCII‐coded communication and image transmission. a,b) Design schematic of encryption/ decryption optical communication system. It demonstrates how the “HIAS” word is converted into an optical signal through ASCII, and the information is encrypted/decrypted according to the XNOR logic operation of the PSPD through the electrical signal key to achieve information security and accurate transmission. c,d) Encrypted image transmission demonstration. A binary image “HIAS” is changed into a series of optical signal sequences, which are encrypted by the electrical signal key through the XNOR operation, in the process of encrypted transmission, only messy image information can be obtained, and the correct information can be obtained only by decrypting again through our sensor.

To extend the utility of the plasmon‐enhanced self‐powered photodetector (PSPD) integrated with reconfigurable optoelectronic logic computations (OELC), we developed a sophisticated encrypted information transmission system, capable of handling both textual and image data with high levels of security, as depicted in **Figure** **5**a. Central to the system operation is the integration of MIR reconfigurable OELC, which utilizes the XNOR gate to facilitate both encryption and decryption processes, ensuring that the transmitted data remains secure from potential interception. The ability to dynamically configure the logic operation in real time, through simultaneous optical and electrical input modulation, represents a significant leap in designing adaptive and secure communication frameworks. In this system, the primary information to be transmitted is encoded using the ASCII, wherein two distinct input signals are generated: the optical signal, which carries the core information (e.g., “HIAS”), and an electrical signal, which contains the encryption key (K). Through the use of reconfigurable optoelectronic logic operations, specifically an XOR logic operation, the optical and electrical inputs are processed concurrently, allowing for the efficient and seamless encryption of the transmitted signal. The signal generator and sourcemeter, under the control of the system's computational unit, orchestrate the input sequences based on both the optical and electrical data, with the output being an encrypted electrical signal that has passed through the XOR logic operation. The encrypted electrical signals are then identified by the LabVIEW‐controlled system, which controls the corresponding pulse sequence of the optical signal based on defined thresholds (e.g., IN1 > 80 nA and IN0 < 80 nA). This modulation enables the generation of a synchronized decryption key (K) sequence, which is subsequently applied to the PSPD. By decoding the output electrical signals via ASCII, the transmitted message “HIAS” is successfully reconstructed. Crucially, the integration of the reconfigurable logic operations within the device ensures that the encrypted information can only be correctly decoded if the appropriate decryption key is applied, thus providing robust protection against interception. Should an unauthorized party attempt to intercept the transmission, their conventional detection methods would only yield a scrambled sequence, as demonstrated in Figure 5b, effectively nullifying any possibility of reconstructing the original information. We perform electrical encryption on the optical signal carrying information and convert the optical signal twice through the encryption/decryption process of the XONR logic operation to achieve secure and accurate transmission of information.

Furthermore, expanding the system application beyond ASCII‐coded text, we demonstrate the capability of the device to perform secure encoded image transmission using a similar encryption protocol, as illustrated in Figure 5c. The image to be transmitted is mapped into a 10 × 10 pixel matrix, with each pixel corresponding to an individual input signal, and the intensity of each pixel representing the magnitude of the signal. These pixel signals are subsequently transformed into temporal sequences, forming a 10‐sequence signal pulse that encodes both the spatial and intensity information of the image. The transmitted signal is encrypted using a predefined binary key sequence (e.g., 1010101010), implemented through XNOR logic operations. The reconfigurable nature of the OELC enables real‐time switching between logic operations, ensuring that the image signal, once encrypted, can only be decrypted using the precise key sequence. If an interception occurs during the transmission process, the decrypted signal will not correspond to the original image, yielding a distorted and unusable reconstruction. The success of this approach in reconstructing the transmitted image (e.g., “HIAS”) accurately, while ensuring that any unauthorized decryption attempts result in meaningless outputs, which is shown in Figure 5d, exemplifies the system's potential for high‐security communication applications.

## 3 Conclusion

We have successfully integrated a Ta_2_NiSe_5_‐based plasmon‐enhanced self‐powered photodetector (PSPD) in the MIR regime, achieving reconfigurable optoelectronic logic operations within a single device. This innovation marks a critical advancement in the field of optoelectronic logic devices, as highlighted in Tables S2 and S3 (Supporting Information), which compares our Ta₂NiSe₅ PSPD OELC with previously reported devices, underscoring its superior responsivity, operational efficiency, and versatility. At the heart of the device functionality lies its precisely engineered plasmonic nanostructures, which not only enhance the absorption of MIR photons but also drive the generation of hot carriers, facilitating efficient photodetection even under zero bias conditions. Through meticulous control of the laser's position and the applied voltage, we configured six fundamental Boolean logic functions, demonstrating the reconfigurable capability of the device for performing real‐time logical computations. By integrating the reconfigurable optoelectronic logic system into a communication framework, we showcased a proof‐of‐concept platform for encrypted data transmission, where the XNOR logic operations play a pivotal role in both the encryption and decryption processes. The work represents a paradigm shift in optoelectronic logic devices, combining plasmonic enhancement, mid‐infrared photodetection, and built‐in encryption into a single, scalable architecture.

## 4 Experimental Section

### Materials Synthesis and Characterization

The Ta_2_NiSe_5_ single crystals, from the collaborator Shanghai Onway Technology Co., Ltd., were synthesized by traditional chemical vapor transportation (CVT) technology. The mixture of Ta, Ni, and Se powders (99.9%, Alfa) in a stoichiometric ratio was thoroughly ground and then combined with 2% mass fraction of iodine as a transport agent. This mixture was loaded into a fused quartz tube, which was then vacuum‐sealed with a pressure >10^−4^ Torr. Subsequently, the quartz tube was placed inside a two‐zone furnace and heated at 750/880 °C for a duration of 10 days. Upon cooling to room temperature, silver strip‐like crystals of Ta_2_NiSe_5_ were obtained. The morphology of Ta_2_NiSe_5_ nanosheets was preliminarily observed by fluorescence microscope (Olympus, OWM‐3M). The micro‐Raman system (Renishaw inVia) with a 532 nm laser was performed to obtain the Raman spectrum of the Ta_2_NiSe_5_. The PL spectra, from Nanjing Metatest Optoelectronics Co., Ltd. were characterized by the confocal microscope (Horiba Scientific LabRAM HR Evolution) and a sequential wavelength 532 nm laser. The Ta_2_NiSe_5_ was analyzed using SEM and EDS spectrum analyzer (Gemini500) to determine its surface topography and elemental composition.

### Device Fabrication and Characterization

The substrate chosen is high‐resistivity silicon with a 300 nm thick SiO_2_ dielectric layer purchased from Shanghai Onway Technology Co., Ltd. The overall nanostructure composed of Cr/Au (10/50 nm) on the substrate surface is patterned using electron beam lithography (EBL, Raith150 Two) and fabricated using high‐vacuum deposition techniques (Ei‐5Z). Ta_2_NiSe_5_ and h‐BN nanosheets are obtained from bulk crystals through mechanical exfoliation using blue tape and then transferred to the center of the overall nanostructure, in turn, to complete the device via a dry transfer method. The preparation process diagram is shown in Figure S5 (Supporting Information).

### Electrical and Optical Characterizations

The morphology and size of the microstructure were observed by scanning electron microscope (SEM, Gemini500). The infrared absorption spectra were obtained using a Fourier Transform Infrared spectrometer (FTIR, Vertex 80) with a microscope (Hyperion 3000). Variable temperature output characteristics are obtained by a low‐temperature probe station (PS‐100) and digital source meter (Keithley 4200‐SCS). The application of bias and concurrent measurement of current and photovoltage were facilitated through the utilization of a highly sensitive dual‐channel digital source meter (Keithley 6482). For fundamental photoelectric testing and performance evaluation, a uniform incident light spot generated by the laser was achieved with spot diameters of 54 µm (1550 nm laser) and 10 µm (4600 nm laser), meticulously focused utilizing different objective lenses. The effective light power density (*P*_eff_) was calculated using *P*_eff_ = *P*_in_ × *A*_device_/*A*_spot_ (*A*_device_ <*A*_spot_) or *P*_eff_ = *P*_in_ (*A*_device_ > *A*_spot_), where the effective photoresponse area of *A*_device_ of PNS and W/OW/O NS are 600 and 400 um^2^, respectively. Photocurrent mapping was conducted by scanning the pressure point console horizontally and vertically under the irradiation of a focused modulated laser, with the photocurrent signal obtained through a lock‐in amplifier (MStarter 200, Metatest). Furthermore, the responsivity (*R*_I_) and external quantum efficiency (*EQE*) under 0.1 V bias are evaluated by the formulas of *R*_I_ = *I*_ph_/*PS* and *EQE* = *hcR*/*qλ* (where *P*, *S*, *h*, *c*, *q*, and *λ* are light intensity, effective irradiated area, Planck constant, speed of light, Elementary charge and incident wavelength). Additionally, noise equivalent power (*NEP*) and normalized detectivity (D*) are the key performance indexes to describe the noise characteristics and sensitivity of photodetectors, which can be calculated by using the following equations: *NEP* = *i*_n_/*R* (*i*_n_ = (2*qI_d_* ∆*f* + 4*k*_B_ *T*∆*f*/*R*)^1/2^) and D* = (*A*·∆*f*)^−1/2^/*NEP*, where*i*_n_, *I_d_*, ∆*f*, *k*_B_, *R* and *A* represent noise current, dark current, bandwidth, Boltzmann constant, resistance and effective area of the device.

### Simulation

The optical numerical simulations in this work were performed using commercial software based on the finite‐difference time‐domain (FDTD) method, Lumerical FDTD solutions. All projects were performed using a TM polarized plane wave incident vertically from the top of the metasurface. The frequency domain power monitor was put on the upper part of the source and the lower part of the structure to calculate the reflection and transmission, respectively, and the frequency domain profile monitor was placed on the structure to monitor the field distribution. A unit cell of the investigated structure was simulated using periodic boundary conditions along the x and y directions and perfectly matched layers along the z direction. A global mesh accuracy of 8 was selected and an extra uniform mesh (5 nm) was added to the metasurface to facilitate rapid convergence of the results. The simulation time was set as 6000 fs with an auto shutoff minimum of 1 × 10^−9^. The refractive indices of Au, Si, and SiO_2_ were derived from Palik's handbook. Visualization of photothermal effects using Lumerical HEAT. With the electric field *E* obtained from the optical simulation, the absorbed power *P* (*P* = 0.5*ωε*_im_|*E*|^2^) of the structure can be derived, which will be further imported into HEAT as a heat source. In the simulation, the mesh is adaptive and the convective boundary conditions at the Au‐air and SiO_2_‐air interfaces are “constant”. The thermal conductivities of Au, air, SiO_2,_ and Si are 316, 0.0263, 1.38, and 148 W mK^−1^ respectively.

## Conflict of Interest

The authors declare no conflict of interest.

## Supporting information



Supporting Information

## Data Availability

The data that support the findings of this study are available from the corresponding author upon reasonable request.
