# Supplementary material for: On‐Chip Metamaterial‐Enhanced Mid‐Infrared Photodetectors with Built‐In Encryption Features
Source: Adv Sci (Weinh). 2025 Jan 10;12(9):2415518. doi: 10.1002/advs.202415518 (PMC11884537; doi:10.1002/advs.202415518)
Supplement: Supplementary file 1 — Supporting Information [file ADVS-12-2415518-s001.docx]

**Supplementary Information**

**On-Chip Metamaterial-Enhanced Mid-Infrared Photodetectors with Built-In Encryption Features**

Shicong Hou^1,2^, Li Han^3^, Shi Zhang^2,4^, Libo Zhang^2^,* Kaixuan Zhang^2^, Kening Xiao^2^, Yao Yang^5^, Yunduo Zhang^2^, Yuanfeng Wen^2^, Wenqi Mo^2^, Yiran Tan^2^, Yifan Yao^1^, Jiale He^2^, Weiwei Tang^2^,* Xuguang Guo^1^,* Yiming Zhu^1^,* Xiaoshuang Chen^2,4^

^1^ Shanghai Key Laboratory of Modern Optical Systems, Terahertz Technology Innovation Research Institute, and Engineering Research Center of Optical Instrument and System, Ministry of Education, University of Shanghai for Science and Technology, 516 Jungong Road, Shanghai 200093, China.

^2^ College of Physics and Optoelectronic Engineering, Hangzhou Institute for Advanced Study, University of Chinese Academy of Sciences, No. 1, Sub-Lane Xiangshan, Xihu District, Hangzhou 310024, China.

^3^College of Optical and Electronic Technology, China Jiliang University, Hangzhou, 310018, China.

^4^State Key Laboratory of Infrared Physics, Shanghai Institute of Technical Physics, Chinese Academy of Sciences, 500 Yu-Tian Road, Shanghai 200083, China.

^5^College of Physics and Optoelectronic Engineering, Shenzhen University, Shenzhen, China.

**^*^**Corresponding author. Email: [zhanglibo@ucas.ac.cn](mailto:zhanglibo@ucas.ac.cn); [tangweiwei@ucas.ac.cn](mailto:tangweiwei@ucas.ac.cn); [xgguo@usst.edu.cn](mailto:xgguo@usst.edu.cn); [ymzhu@usst.edu.cn](mailto:ymzhu@usst.edu.cn).

**This Supplementary Information includes:**

Notes S1 to S3

Figures. S1 to S14

Tables S1and S2

References

**Note S1:** **Simulation of plasmon resonance unit and integral structural design**

For plasmon at flat, smooth metal-insulator interface, the dispersion relation can be written as

$k_{sp}=\frac{\omega}{c_{0}}\sqrt{\frac{\varepsilon_{d}\varepsilon_{m}}{\varepsilon_{d}+\varepsilon_{m}}}$ (S1)

where *c*_0_ is the vacuum speed of light, and $\varepsilon_{d}$ and $\varepsilon_{m}$ are the dielectric functions of the medium and metal, respectively. Since the metal is a lossy material in the considered wavelength, $k_{sp}$ has an imaginary part, i.e., $k_{sp}=k_{1}+ik_{2}$. In the Drude interval, an additional wave vector is required to compensate for this gap since $k_{sp}$ is always on the right side of the incident wave vector $k_{sp}$. Preparing a periodic structure is an effective way to excite plasmon with a period $\Lambda$ which can be written as

${\frac{2\pi}{\Lambda}=k}_{1}-k_{0}sin\theta$ (S2)

where $\theta$ is the angle of incident light. The numerical relationship between the resonance wavelength $\lambda_{res}$ and the width of the structure can be obtained by further calculations, i.e., $\lambda_{res}\propto A\sqrt{w}$ where *A* is a dimensionless constant and *w* is the width of the structure.

Then, in order to determine the specific structural parameters of the plasmon resonance unit, we simulate the light absorption rate determined by the incident wavelength and the structure width, and also simulate the function of the light absorption rate with the incident wavelength and polarization, the simulation results are shown in Figure S1a and b. In order to achieve the resonance enhancement of MIR with a target wavelength of 4600 nm, we determine the structure size such as Figure S2a and Table S1. However, to elucidate the amplifying impact of the plasmon resonance unit, we designed an interdigital structure without the plasmon metal structure on the other half, which can not only compare the absorption characteristics of the two structures, but also realize self-powered photothermoelectric detection with the unique asymmetric structure, the structure and its corresponding size can be see in Figure S2b and Table S1.


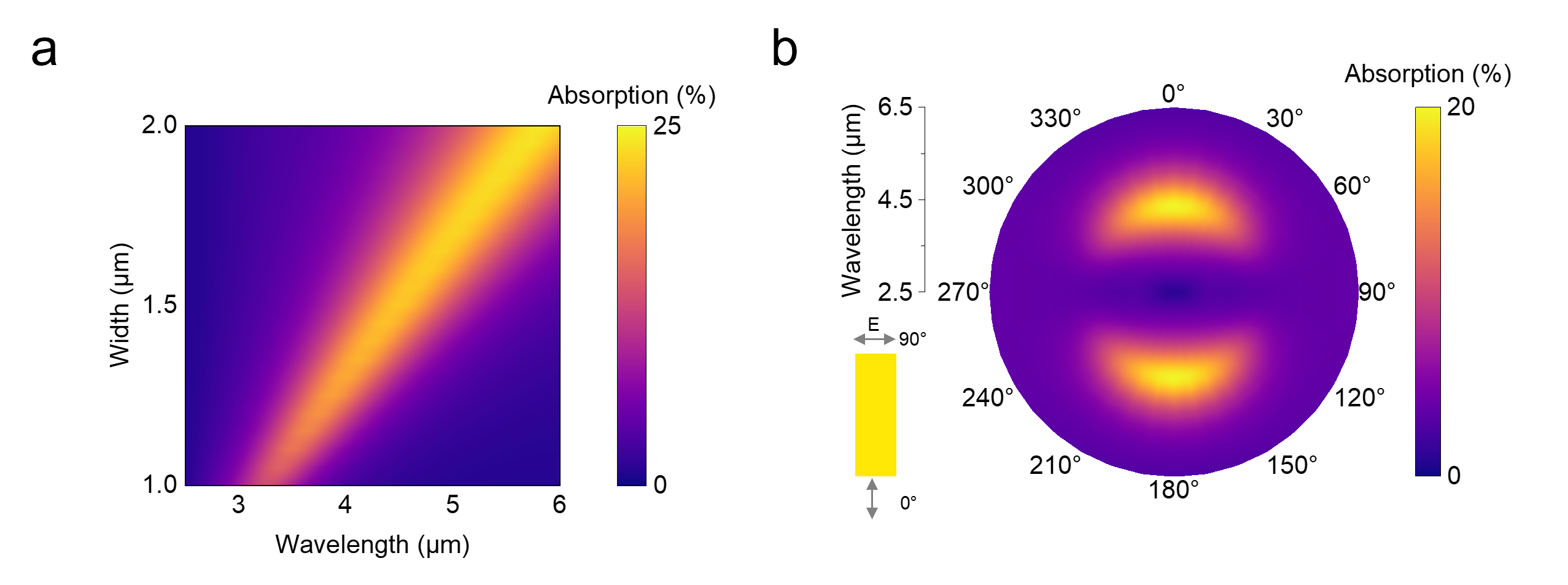
 **Figure S1: Optical absorption** **simulation based on FDTD. a**, wavelength and structure width dependent absorption by simulation. **b**, simulated absorption as a function of wavelength and optical polarization.

**
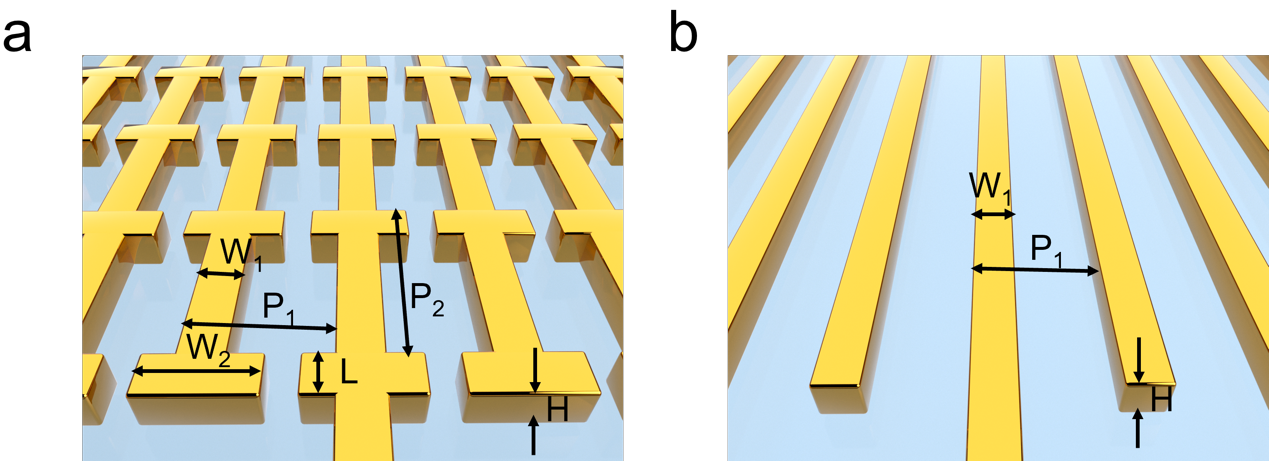
**

**Figure** S**2: Structure Size marking of the PNS and GNS. a**, In the PNS, the transverse and longitudinal periods of the dipole P_1_ and P_2_, length, width and thickness of dipole L, W_2_ and H, width of metal nanowires connecting dipoles W_1_. **b**, In the GNS, The period, width and thickness of the interdigital metal structure P_1_, W_1_ and H.

**Table S1: Structure size parameters of the PNS and INS.** The period, thickness, length and width of the nanostructure.

|  | P_1_ (µm) | P_2_ (µm) | W_1_ (µm) | W_2_ (µm) | L (µm) | H (nm) |
| --- | --- | --- | --- | --- | --- | --- |
| PNS | 2.5 | 2.5 | 0.2 | 1.6 | 0.1 | 60 |
| GNS | 2.5 |  | 0.2 |  |  | 60 |

**Note S2: Preparation and characterization of** **the PDSD based on Ta_2_NiSe_5_**

**Preparation of metal nanostructures:** The preparation process of the PDSD based on Ta_2_NiSe_5_ is shown in Figure S5. Firstly, we spin-coated 300 nm PMMA (950K-A4) on a clean Si/SiO_2_ substrate, and then exposed the structural pattern of our design through EBL. After 30 s of development with developer (AR600-56) and isopropanol cleaning, the designed pattern was displayed on PMMA. Subsequently, Cr/Au (10/50 nm) was evaporated on PMMA by high vacuum deposition technology, and then the excess metal was removed by acetone using lift-off technology to obtain the metal nanostructures we virtualized. The morphology was observed by optical microscopy and scanning electron microscopy as shown in Figure S3a-S3c. In addition, we have employed atomic force microscopy (AFM) to meticulously characterize the morphology and planarity of the gold microstructure, thereby ascertaining the formation of a uniform gold microstructure. The ensuing data, as delineated in the Figure S3d, reveal that the heights of the gold microstructure units in the lateral and vertical directions are 63.5 nm and 62.7 nm, respectively, with a relatively uniform height distribution, thereby demonstrating that the gold microstructure were uniformly and densely deposited on the substrate.

**Characterization of photoresponse materials:** Ta_2_NiSe_5_ is bound together by feeble van der Waals interactions between its layers and showcases a periodic sawtooth chain configuration comprising two TaSe_6_ octahedral single chains and NiSe_4_ tetrahedral single chains^1^, the material has been proven to have good photoelectric properties in various bands. Here, we used Raman spectroscopy to characterize the molecular vibration modes of Ta_2_NiSe_5_ before and after Au integration. Figure S4b shows that Ta_2_NiSe_5_ does not form new bonds upon contact with Au, nor does it undergo a modification of its bandgap. PL spectroscopy excited at 532 nm to reflect the vibration mode and electron transition process of Ta_2_NiSe_5_, the results are shown in the Figure S4c, which is consistent with previous studies. In order to verify the composition elements of the material, we carried out SEM and EDS analysis, tthe results show that it consists of Ta, Ni and Se elements (see Figure S7). In addition, the thermoelectric properties of Ta_2_NiSe_5_ are also realized by measuring its temperature-dependent output characteristics, and its conductance increases nonlinearly with the increase of temperature, which is shown in Figure S4a.

**Completion of the device and characterization of photoelectric properties:** As the optical functional material Ta_2_NiSe_5_ and the protective layer h-BN are successively transferred to the top of the metal nanostructure by dry transfer, the device is completed. At the same time, we also fabricated a Ta_2_NiSe_5_ device without nanostructure to compare the photoelectric properties. Firstly, the SPCM was utilized to determine the response peak values of the two structures (the scanning area is displayed in Figure S6. The results indicated that the photoresponse of the PNS was more than 10 times higher than that of the structure w/o NS. However, the measured absorption rate of the PNS increased by only 14%, there must be other influencing factors at play. Analysis revealed that, compared to the traditional electron transition which requires overcoming the bandgap limitation, hot electrons excited by plasmonics only need to cross the Schottky barrier to achieve transition (where the Schottky barrier between Ta_2_NiSe_5_ and Au is smaller than the bandgap of Ta_2_NiSe_5_), this process is illustrated in Figure S8. To better illustrate the enhancement effects of the PNS, we identified its optimal photoresponse position through the photocurrent scanning results of PNS, INS, and ONS (Figure S9). Building upon this, we analyzed the photocurrent of different structures from the visible to the MIR spectrum (see Figure S10). To demonstrate the stability of the device, we analyzed the dependence of the photocurrent on incident power under various bias voltages for different structures, the test results are presented in Figure S11. The results indicate that as the bias voltage changes, the photocurrent of the device consistently maintains a good linear relationship with the incident power, suggesting that the photocurrent of the device can be effectively modulated. A comparative study revealed that the photoresponse of PNS in the target wavelength is significantly enhanced. Due to the hot electron transfer speed excited by plasma being on the fs scale, it is theoretically capable of significantly enhancing the response speed of materials. To investigate this, we tested the square wave signals of devices based on w/o NS and PNS under MIR pulses, as shown in Figure S12. To implement the photoelectric logic computing based on PSPD, we demonstrated four distinct states of light input, with their corresponding output characteristics displayed in Figure S13.

**
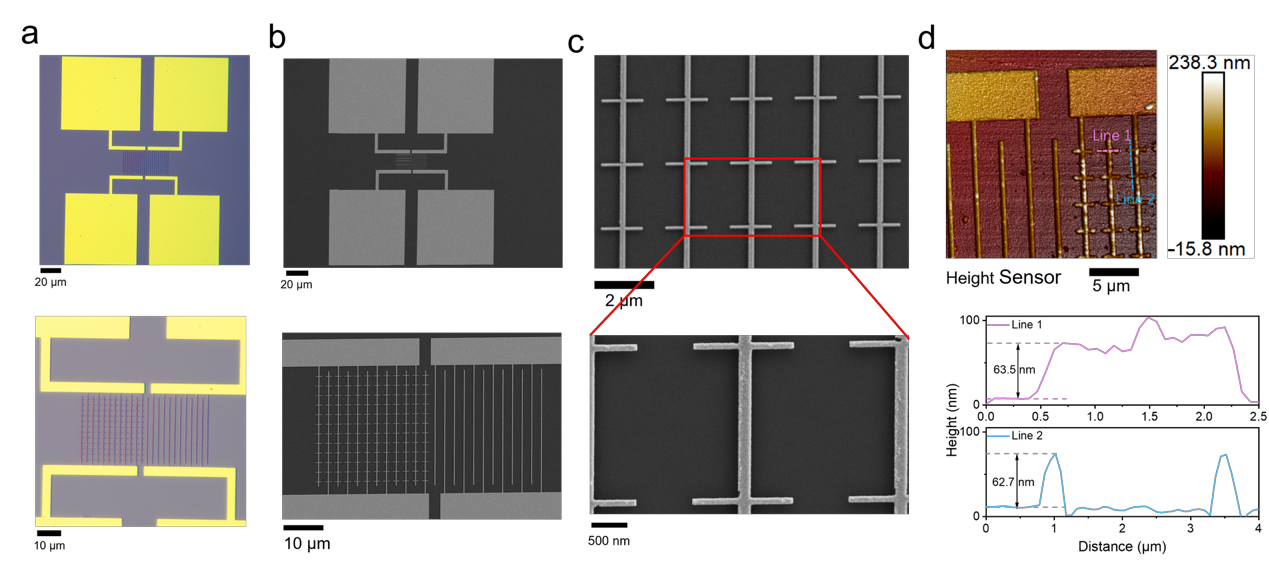
**

**Figure S3: Morphology characterization of metal nanostructure at different magnifications. a**, optical microscope photographs on a scale bar of 20 µm and 10 µm. **b**, SEM image on a scale bar of 20 µm and 10 µm. **c**, SEM image on a scale bar of 2 µm and 500 nm **d**, AFM image and height of the Au microstructure.


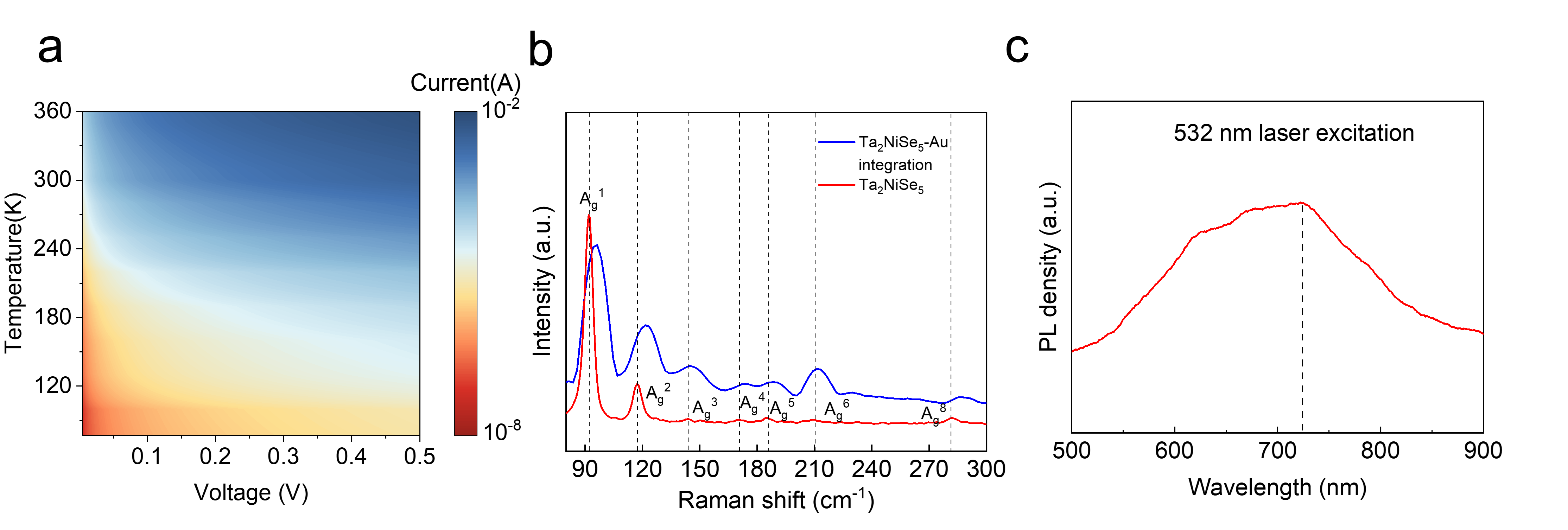


**Figure S4: Characterization of** **Ta_2_NiSe_5_ two-dimensional materials. a**, Temperature dependent output characteristic. **b**, Raman spectrum of Ta2NiSe5before or after the integration of Au. **c**, photoluminescence spectra of Ta_2_NiSe_5_ nanosheets.


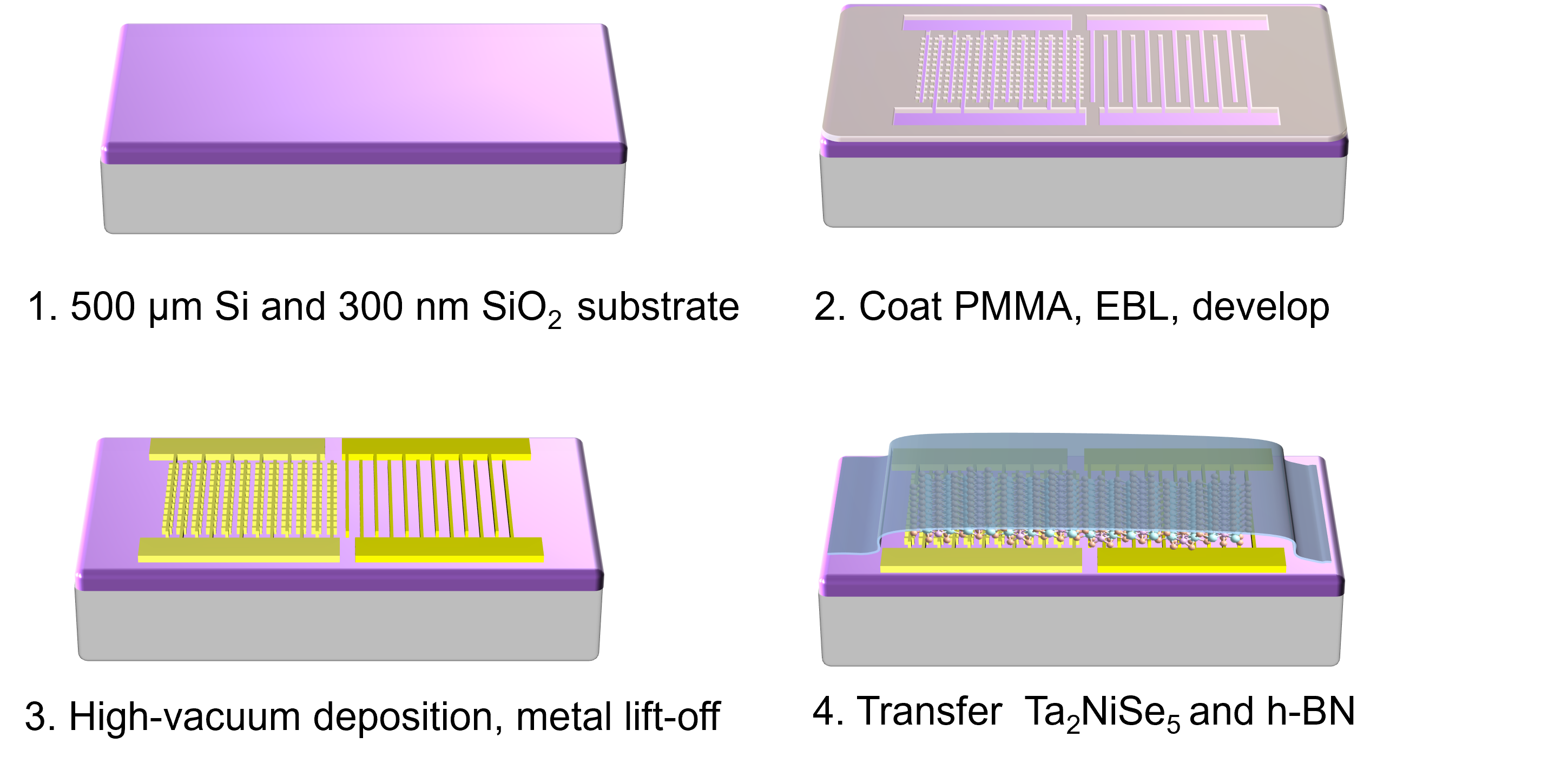


**Figure S5: Preparation process of metal nanostructure**

**
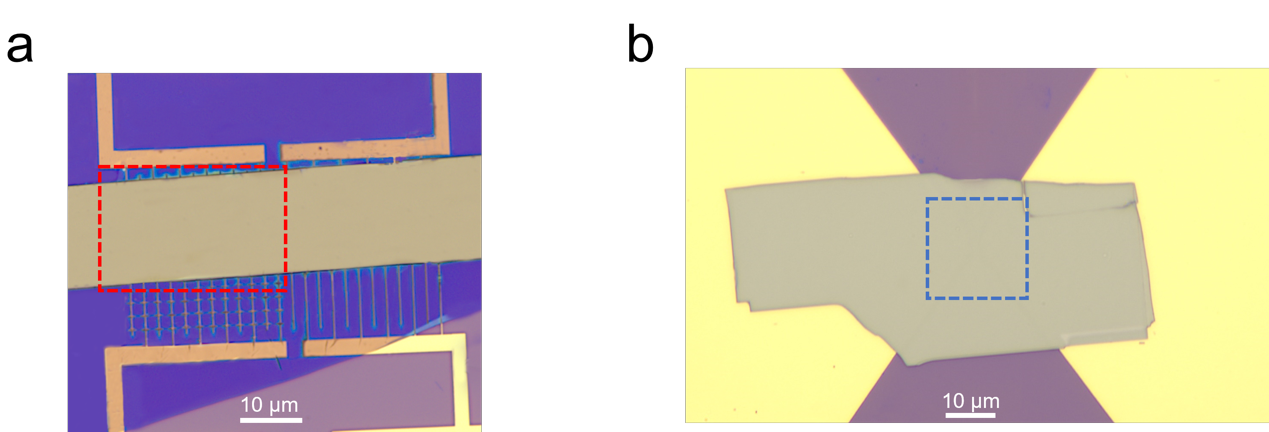
 Figure S6: Optical micrographs of the devices of PNS and W/O NS. a**, **b**, partial enlarged detail of PNS and W/O NS on the scale bar of 10 µm, the red and blue dotted wireframes correspond to their SPCM regions, respectively.


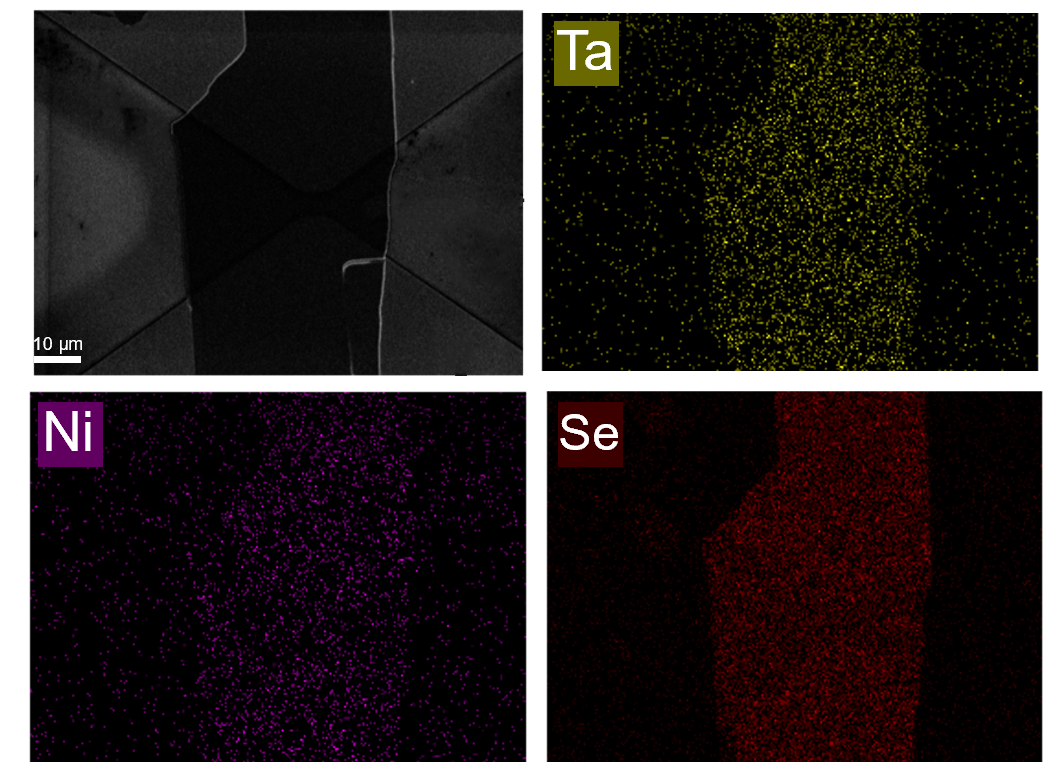
 **Figure S7: SEM image and Elemental analysis by energy dispersive spectrometry of the** **Ta_2_NiSe_5_ nanosheet.**

**
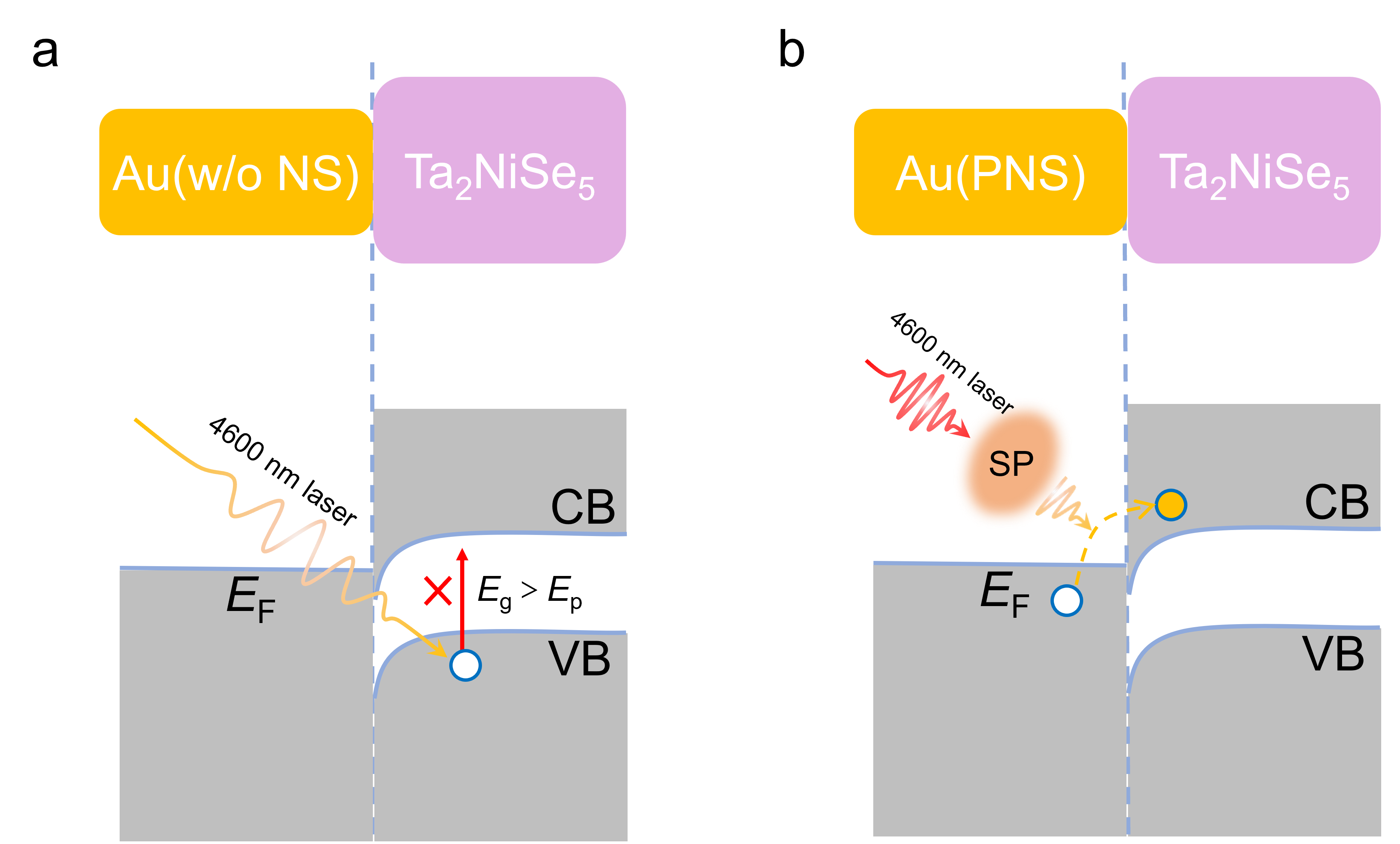
 Figure S8: The energy band diagram of Ta_2_NiSe_5_ under MIR excitation after contact with different metal structures. a**, Upon Ta_2_NiSe_5_ contact with Au (W/O NS), electrons are unable to overcome the bandgap to achieve transition. **b**, Upon Ta_2_NiSe_5_ contact with Au (PNS), electrons can overcome the Schottky barrier and transition to the conduction band.

**
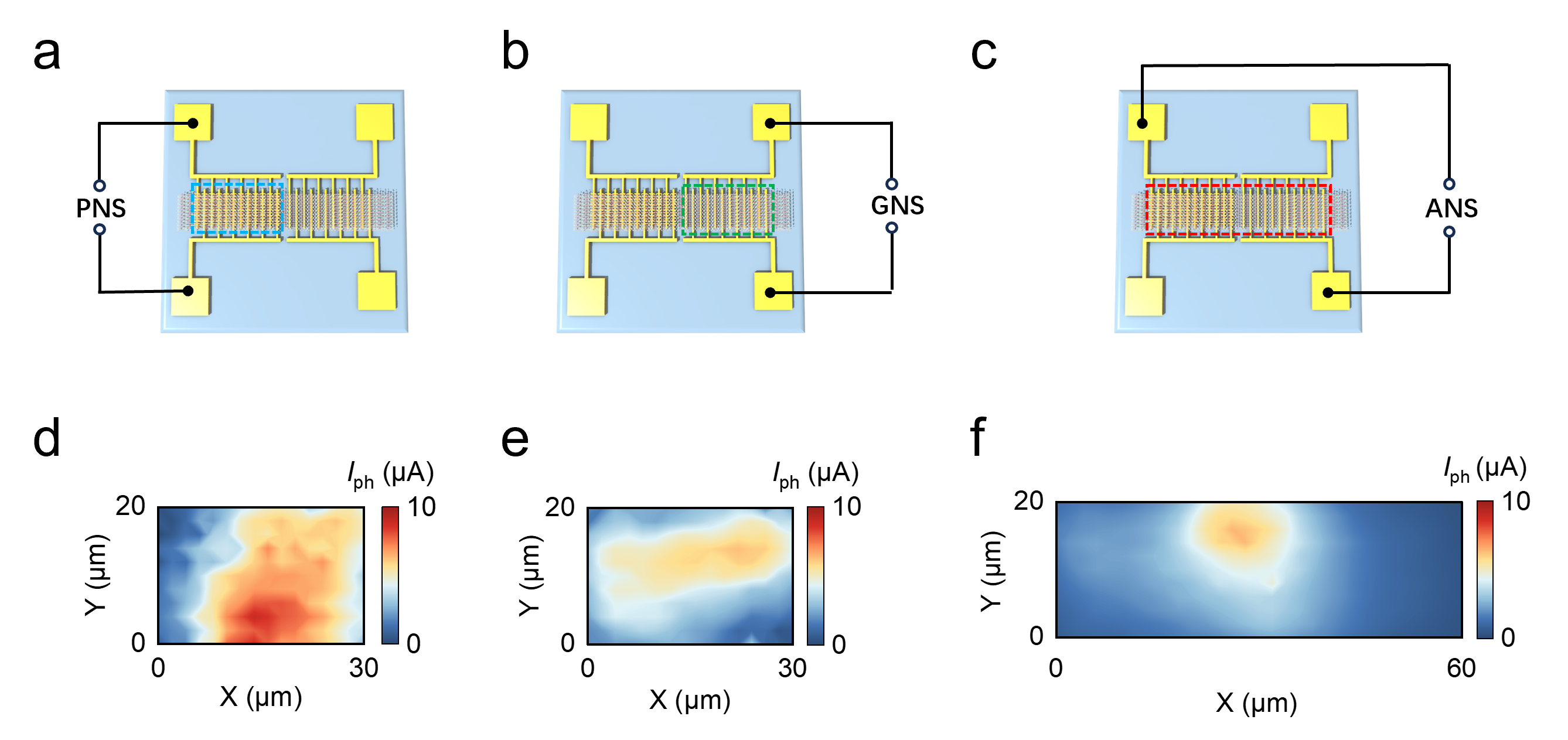
 Figure S9: The SPCM of different metal nanostructures. a-c**, The testing diagram of the circuit designs with PNS, GNS and ANS is illustrated, where the dashed lines denote the respective SPCM areas. **d-f**, SPCM results of PNS, GNS and ANS under 4600 nm laser at 0.1 V bias.


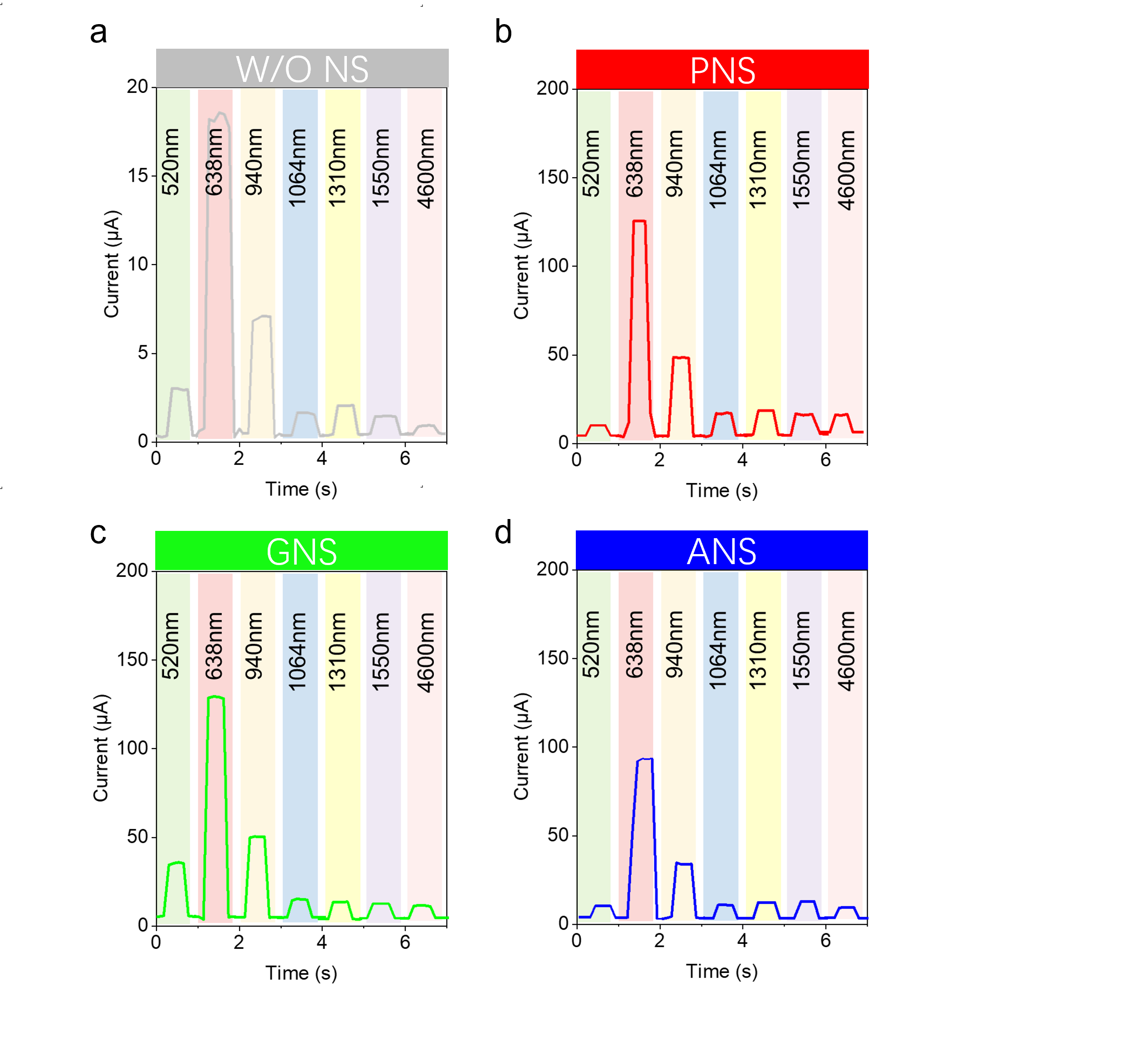


**Figure S10: The temporal photoresponse of the various structures. a-d**, the temporal photoresponse of W/O NS, PNS, GNS and ANS under 0.1V bias across a broad wavelength range of 520 nm, 638 nm, 940 nm, 1064 nm, 1310 nm, 1550nm and 4600 nm.

**
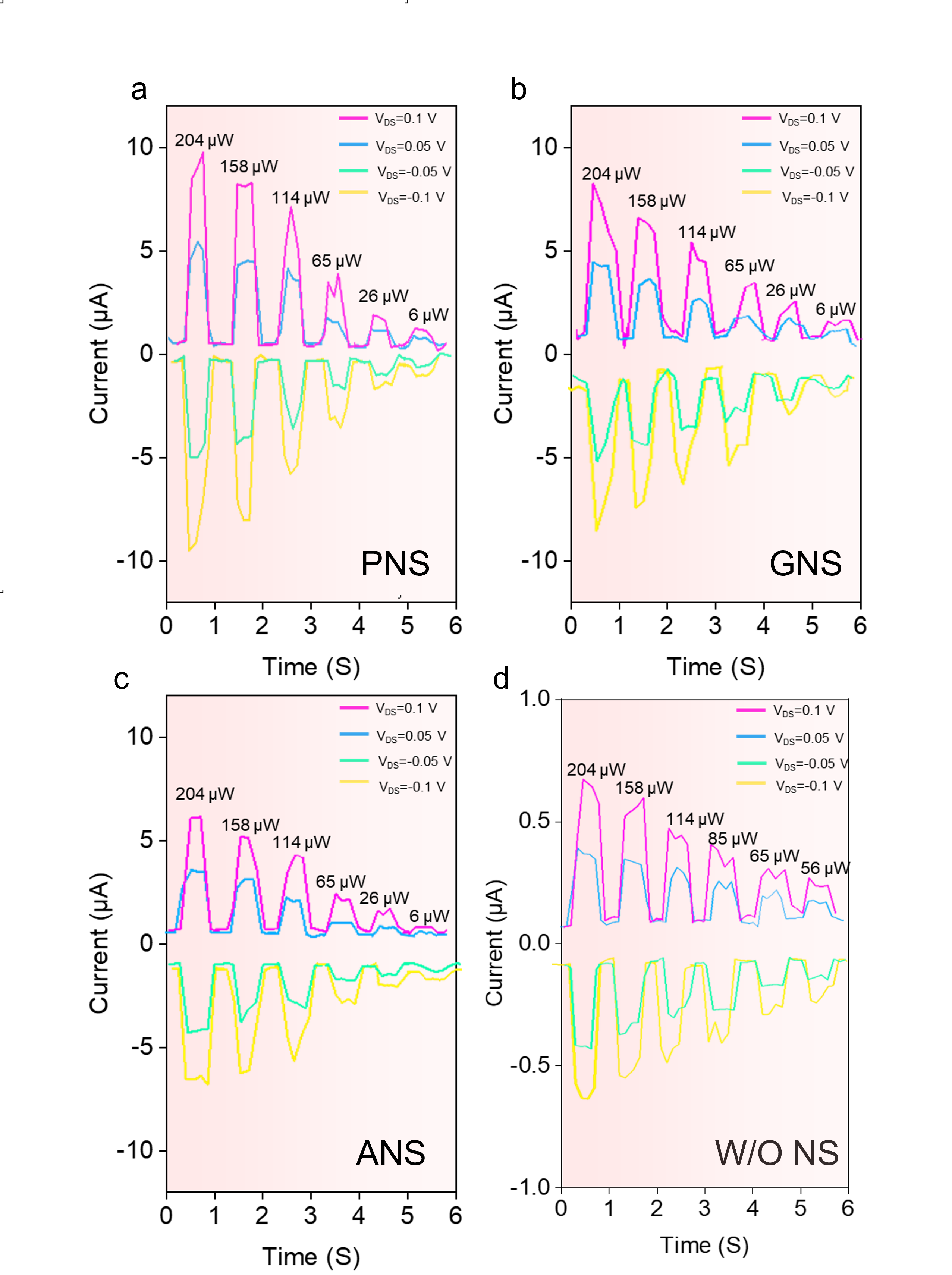
 Figure S11: The output curves of the optimal photoresponse positions for various structures under on-off of MIR. a-d**, Under 4600nm laser irradiation, the I-T curves of PNS, GNS, ANS and W/O NS are characterized as the bias voltage varies from -0.1 to 0.1V and the incident power ranges from 6 to 204 μW.


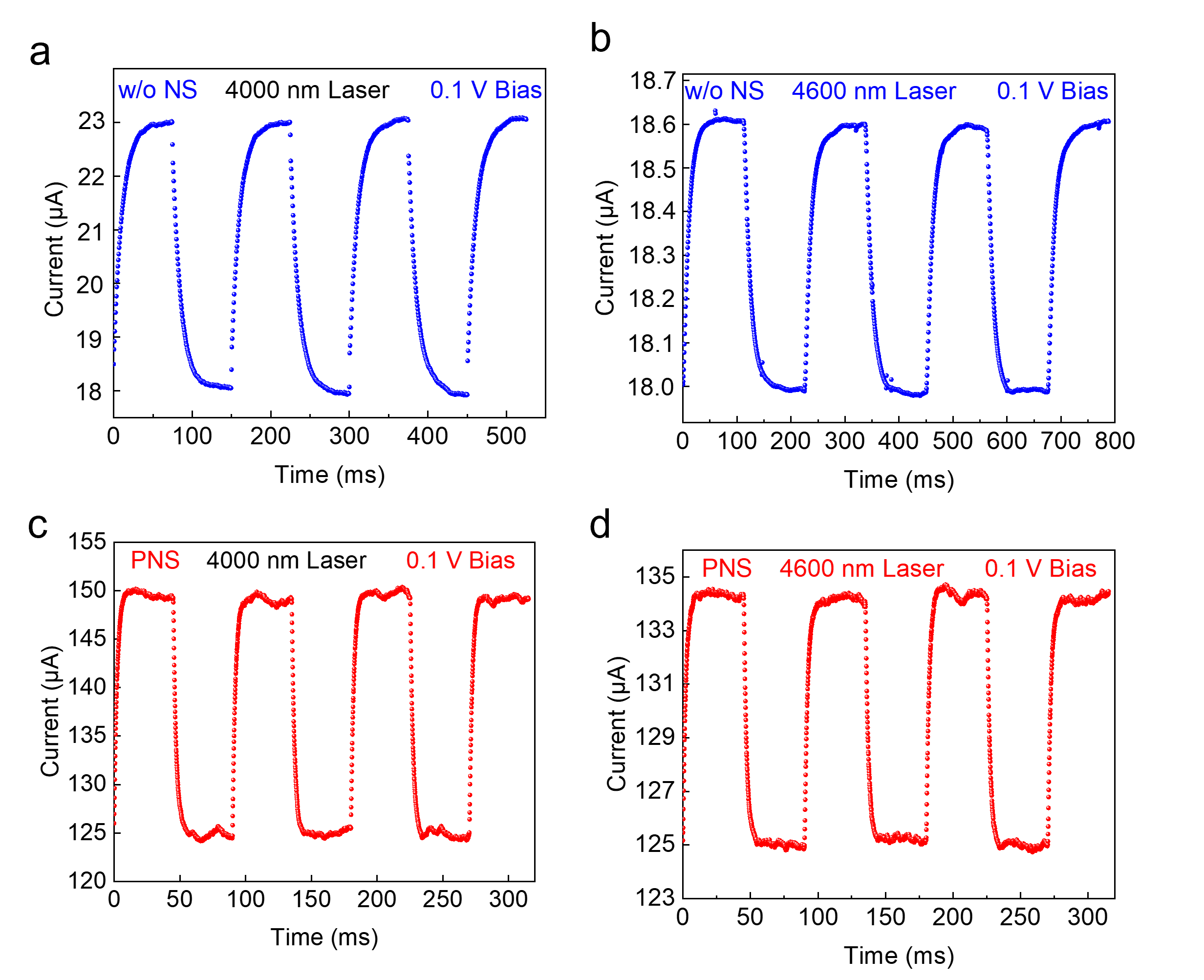


**Figure S12: The output current of Ta_2_NiSe_5_ devices during the on-off cycles of the MIR laser. a**, **b**, Time-resolved photocurrents of w/o NS at 4000nm and 4600nm under 0.1v bias. **c**, **d**, Time-resolved photocurrents of PNS at 4000nm and 4600nm under 0.1v bias.


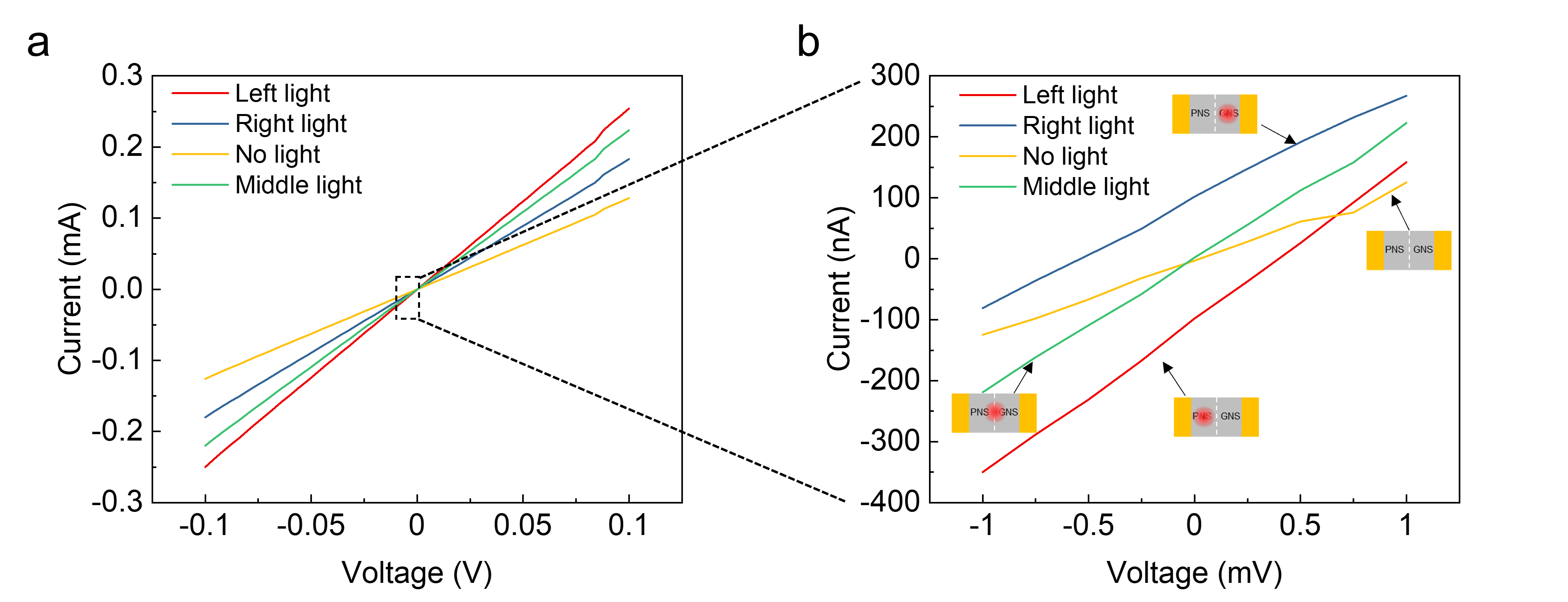


**Figure S13: The output characteristic of MIR radiation at different positions on nanostructures and without incident laser. a**, The I-V curves of the Ta_2_NiSe_5_ device based on ANS under different 4600 nm laser states are presented. **b**, Local enlargement of Fig. a, the various states of the laser are depicted in the accompanying illustrations.

**Note S3:** **NOR, XOR and NAND** **Logic computings design**

In this study, we show a total of six logic computings: AND, OR, NAND, NOR, XOR and XNOR. The design ideas of NOR, XOR and NAND logic computings are as follows (see Figure S14):

**NOR:** “0” and “1” states of IN1are set as the on and off conditions of the MIR laser located at the center of the PNS, respectively. The “0” and “1” states of IN2 correspond to 0 mV and 0.5 mV biases, respectively. Among the input sequences IN00, IN01, IN10, and IN11, a significant photocurrent (> 80 nA) is produced only when there is no bias and the structure is irradiated by mid-infrared light, corresponding to an OUT-1 state, thus implementing a logical NOR computing.

**XOR:** In the case of IN1, “1” and “0” are set to correspond to the activation and deactivation of the mid-infrared laser at the PNS, respectively. For IN2, “1” and “0” correspond to the same states at the INS. The device outputs obvious photocurrent only when one of the light sources is active, which corresponds to OUT-1. Therefore, with input sequences of IN00, IN01, IN10, and IN11, the output signals are OUT-0, OUT-1, OUT-1, and OUT-0, respectively, realizing a logical XOR computing.

**NAND:** “1” and “0” of IN1 correspond to the MIR laser located at the center of the ONS and in the PNS, respectively. IN2's “1” and “0” represent the bias of 0 mV and -0.5 mV, respectively. The photocurrent will be very weak (less than 80 nA) only when the laser is at the center of the ONS under a 0 bias state, achieving an output of 0. Thus, when inputting four types of sequences, the output will be the result of a logical NAND computing.


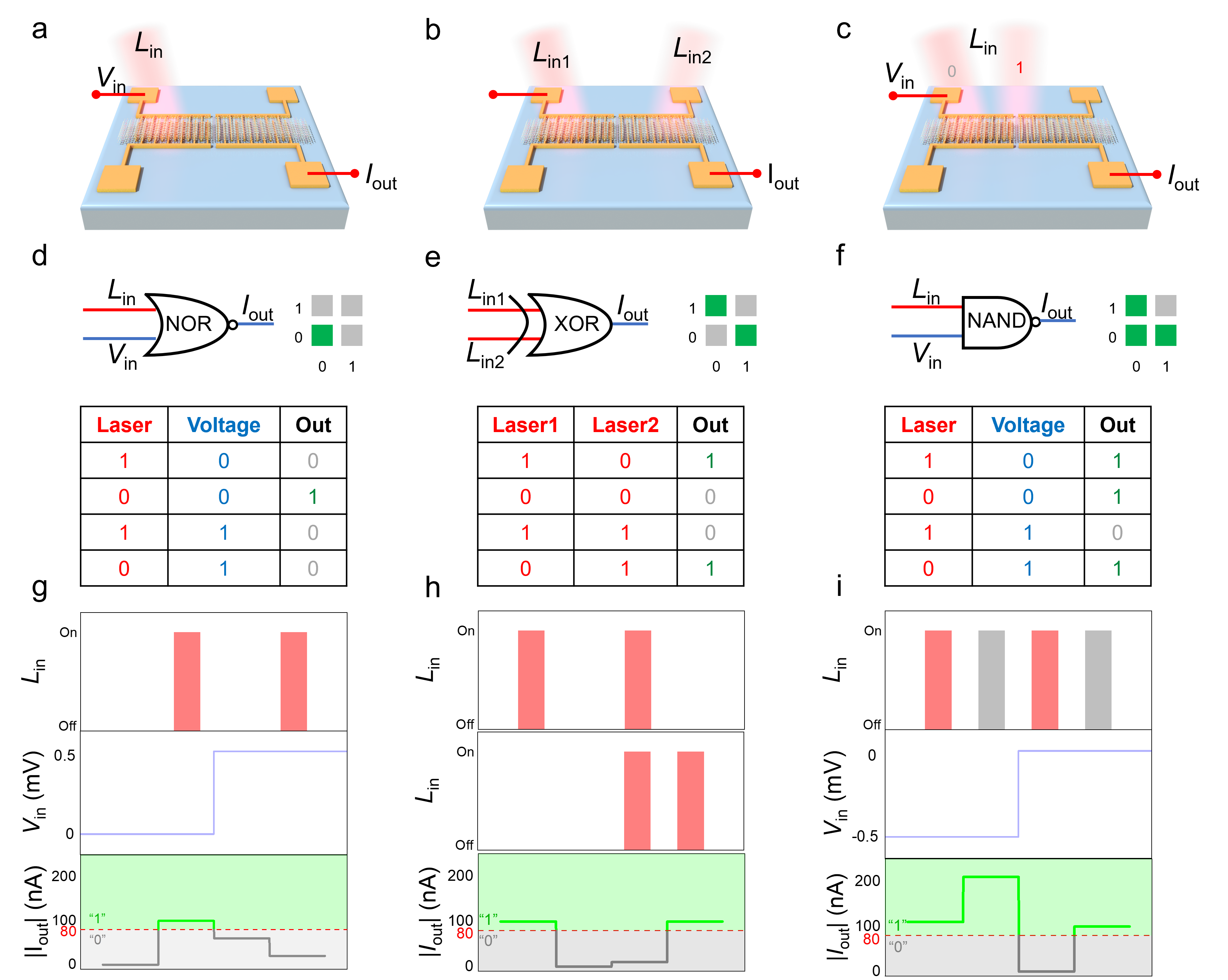
 **Figure S14: Characterization of MIR OELCs based on PSPD. a-c**, Schematics of the logic NOR, XOR and NAND computing system with mixed optical and electrical inputs. **d-f**, Symbolic schematics and truth tables of the logic NOR, XOR and NAND computing. **g-i**, The absolute value of the output current measured under the input sequence corresponding to the logic NOR, XOR and NAND computing.

**Table S2: Performance comparison between Ta_2_NiSe_5_** **PSPD** **OELC and reported optoelectronic logic devices.**

| Materials | Wavelength (nm) | Bias (V) | responsivity | No. of OELC | Self-powered detection | Ref. |
| --- | --- | --- | --- | --- | --- | --- |
| WSe_2_/h-BN/Al_2_O_3_ | 405 | 1 | / | 3 | no | 2 |
| BaTiO_3_/LaNiO_3_ | 405/785 | 0 | / | 5 | yes | 3 |
| CsPbX_3_ PQDs | 430/530 | 0.1 | / | 4 | no | 4 |
| WSe_2_/h-BN/SiO_2_ | 275/532 | 0 | / | 4 | yes | 5 |
| CSi-PSi | 658 | 5 | 21mA/W | 4 | no | 6 |
| CdSe-ZnS QDs | 635 | 5 | 10.7 mA/W | 3 | no | 7 |
| CuInS_2_-TiO_2_ | 365/475 | 0 | 44.8 mA/W | 1 | yes | 8 |
| Perovskite | 625/940 | 0 | 8.7 mA/W | 5 | yes | 9 |
| Ta_2_NiSe_5_ | 4600 | 0.1 | 47 mA/W | 6 | yes | This work |

**Table S3:** **Performance parameters compared with previous plasmonic works**

| Description | Mechanism | Wavelength (μm) | Responsivity | NEP  (nW/Hz^1/2^) | Detectivity  (Jones) |
| --- | --- | --- | --- | --- | --- |
| Metasurface-pyroelectric AlN  Detector ^[10]^ | PyE | 0.65-2 | 0.18 V/W | 679 | 3.9×10^5^ |
| nanoantenna-mediated semimetal photodetector ^[11]^ | PTE | 4.0 | 15.6 V/W |  | 1.56×10^6^ |
| Plasmon induced graphene  detector ^[12]^ | PTE | 0.65,0.75 | 0.125 mA/W |  |  |
| Graphene pyroelectric bolometer ^[13]^ | PyE & PC | 7.5-10 | 0.23 mA/W | 500 | 6×10^4^ |
| PdSe2/Au metamaterials ^[14]^ | PTE | 5.3 | 3.6 V/W | 9.7 | 2.5×10^5^ |
| Metasurface-mediated graphene  detector ^[15]^ | BPVE | 4 | 27 V/W | 0.124 |  |
| metamaterial-enhanced Ta₂NiSe₅ photodetector（this work） | PTE | 0.52-4.6 | 46mA/W  36.8 V/W | 0.239 | 1.02×10^7^ |

The abbreviations of mechanisms represent photo-thermoelectric (PTE), pyroelectric (PyE), photoconducting (PC) and bulk photovoltaic (BPVE) effects. The wavelength denotes the working range of wavelength. NEP means noise equivalent power.

**Supplementary References**

1. C. L. Tan, P. Y, Y. L. Hu, J. Z. Chen, Y. Huang, Y. Q. Cai, Z. M. Luo, B. Li, Q. P. Lu, L. H. Wang, Z. Liu and H. Zhang. *J. Am. Chem. Soc.* **2015**, *137*, 10430.
2. S. Wang, X. Pan, L. Lyu, C.-Y. Wang, P. Wang, C. Pan, Y. Yang, C. Wang, J. Shi, B. Cheng, W. Yu, S.-J. Liang, F. Miao, *ACS Nano* **2022**, *16*, 4528.
3. H. Dan, H. Li, L. Xu, C. Guo, C. R. Bowen, Y. Yang, *InfoMat* **2024**, *6*, e12531
4. J. Pei, X. Wu, W.-J. Liu, D. W. Zhang, S.-J. Ding, *ACS Nano* **2022**, *16*, 2442.
5. F. Gong, W. Deng, Y. Wu, F. Liu, Y. Guo, Z. Che, J. Li, J. Li, Y. Chai, Y. Zhang, *Nano Res.* **2024**, *17*, 3113.
6. J. Kim, H.-C. Lee, K.-H. Kim, M.-S. Hwang, J.-S. Park, J. M. Lee, J.-P. So, J.-H. Choi, S.-H. Kwon, C. J. Barrelet, H.-G. Park, *Nat. Nanotechnol.* **2017**, *12*, 963.
7. B. J. Kim, N.-K. Cho, S. Park, S. Jeong, D. Jeon, Y. Kang, T. Kim, Y. S. Kim, I. K. Han, S. J. Kang, *RSC Adv.* **2020**, *10*, 16404.
8. M. H. Li, J. P. Xu, K. F. Zhu, S. B. Shi, Q. Y. Zhang, Y. C. Bu, J. Chen, J. H. Xu, Q. Zheng, Y. J. Su, X. S. Zhang, L. Li, *J. Mater. Chem. C* **2021**, *9*, 14613.
9. W. Kim, H. Kim, T. J. Yoo, J. Y. Lee, J. Y. Jo, B. H. Lee, A. A. Sasikala, G. Y. Jung, Y. Pak, Perovskite multifunctional logic gates via bipolar photoresponse of single photodetector. *Nat. Commun.* **2022**, *13*, 720.
10. J. W. Stewart, J. H. Vella, W. Li, S. Fan, M. H. Mikkelsen, *Nat. Mater.* **2020**, *19*, 158.
11. J. Wei, C. Xu, B. Dong, C.-W. Qiu, C. Lee, *Nat. Photon.* **2021**, 15, 614.
12. V. Shautsova, T. Sidiropoulos, X. Xiao, N. A. Güsken, N. C. G. Black, A. M. Gilbertson, V. Giannini, S. A. Maier, L. F. Cohen, R. F. Oulton, Nat. Commun. **2018**, *9*, 5190.
13. U. Sassi, R. Parret, S. Nanot, M. Bruna, S. Borini, D. De Fazio, Z. Zhao, E. Lidorikis, F. H. L. Koppens, A. C. Ferrari, A. Colli, Nat. Commun. **2017**, *8*, 14311.
14. M. Dai, C. Wang, B. Qiang, F. Wang, M. Ye, S. Han, Y. Luo, Q. J. Wang, Nat. Commun. **2022**, *13*, 4560.
15. J. Wei, C. Xu, B. Dong, C.-W. Qiu, C. Lee, *Nat. Photon.* **2021**, *15*, 614.
